# Cerium oxide nanoparticles display antilipogenic effect in rats with non-alcoholic fatty liver disease

**DOI:** 10.1038/s41598-019-49262-2

**Published:** 2019-09-06

**Authors:** Silvia Carvajal, Meritxell Perramón, Denise Oró, Eudald Casals, Guillermo Fernández-Varo, Gregori Casals, Marina Parra, Bernardino González de la Presa, Jordi Ribera, Óscar Pastor, Manuel Morales-Ruíz, Víctor Puntes, Wladimiro Jiménez

**Affiliations:** 1 Biochemistry and Molecular Genetics Service, Hospital Clínic Universitari, IDIBAPS, CIBERehd, Barcelona, Spain; 2 School of Biotechnology and Health Sciences, Wuyi University, Jiangmen, 529020 China; 3 Department of Biomedicine, University of Barcelona, Barcelona, Spain; 4 Working group for the biochemical assessment of hepatic disease-SEQCML, Barcelona, Spain; 5 Clinical Biochemistry Service, Hospital Universitario Ramón y Cajal-IRYCIS, Madrid, Spain; 6 Institut Català de Recerca i Estudis Avançats, (ICREA), Barcelona, Spain; 7 Vall d’Hebron Institute of Research (VHIR), Barcelona, Spain; 8 Institut Català de Nanociència i Nanotecnologia (ICN2), Bellaterra, Spain

**Keywords:** Nanoparticles, Biologics, Non-alcoholic fatty liver disease

## Abstract

Non-alcoholic fatty liver disease (NAFLD) is the most common cause of chronic liver disease worldwide, ranging from steatosis to non-alcoholic steatohepatitis (NASH). Recently, cerium oxide nanoparticles (CeO_2_NPs) have emerged as a new antioxidant agent with hepatoprotective properties in experimental liver disease. The aim of the current investigation was to elucidate whether CeO_2_NPs display beneficial effects in an experimental model of NAFLD.Therefore, fifteen Wistar rats were subjected to a methionine and choline deficient diet (MCDD) for 6 weeks and intravenously treated with CeO_2_NP or vehicle during the weeks three and four of the diet. The effect of CeO_2_NPs on serum biochemistry, hepatic steatosis, inflammation, fatty acid content and expression of reactive oxygen species (ROS) and lipid metabolism related genes was assessed. MCDD fed rats showed increased inflammation, enhanced hepatic lipid accumulation of both saturated and unsaturated fatty acids (FAs) and overexpression of genes related to fatty liver and ROS metabolism. Treatment with CeO_2_NPs was able to reduce the size and content of hepatocyte lipid droplets, the hepatic concentration of triglyceride- and cholesterol ester-derived FAs and the expression of several genes involved in cytokine, adipokine and chemokine signaling pathways. These findings suggest that CeO_2_NPs could be of beneficial value in NAFLD.

## Introduction

Non-alcoholic fatty liver disease (NAFLD) is the most common cause of chronic liver disease in the world with a prevalence of 20–40% in the general population and up to 95% in subjects with obesity and diabetes^1,2^. NAFLD is characterized by an abnormal accumulation of fatty acids inside the hepatocytes and includes a broad spectrum of liver diseases, ranging from mild to severe steatosis and non-alcoholic steatohepatitis (NASH)^3^. In turn, hepatocellular lipid accumulation along with liver inflammation, oxidative stress and apoptosis are the main characteristics of NASH^4,5^. Both NAFLD and NASH have the potential to evolve into liver fibrosis and cirrhosis. In addition, these diseases increase the risk of developing hepatocellular carcinoma (HCC), which can be related to cirrhosis or arise in the steatotic liver without evidence of underlying cirrhosis^3,6^.

Oxidative stress is considered a key pathogenic mechanism involved in the progression from steatosis to NASH^7–9^. In humans and experimental animal models of NASH, lipotoxicity plays an essential role in cell death and in the generation of oxidative stress-related products^10,11^. Lipid accumulation along with high levels of circulating free fatty acids induce mitochondria structural and functional abnormalities, leading to increased reactive oxygen species (ROS) production and apoptosis^12^. Also, elevated expression and activity of hepatic CYP2E1 has been observed in human and animal models of NASH, representing a potent source of ROS^13^. Moreover, lisosomal permeabilization induced by FFA exposure plays an important role in apoptosis and endoplasmic reticulum (ER) stress^14^. The high levels of ROS further induce oxidative stress with the subsequent activation of inflammatory and profibrogenic pathways^15,16^. Despite cellular metabolism is dominated by redox-based processes, targeting of the cellular redox sensitive pathways—redoxome—is still an uncommon therapeutic practice^17^. During the last few years antioxidant substances, such as superoxide dismutase (SOD), resveratrol, colchicine, eugenol or vitamins E and C raised increasing interest as potential therapeutic agents in chronic liver diseases^18–20^. These substances have demonstrated their efficacy in reequilibrating hepatic ROS metabolism and thereby improving liver functionality^19,20^. However, despite much enthusiasm in the 1980s and 1990s, many well-known agents have not successfully passed the scrutiny of clinical trials for prevention and treatment of various diseases^21^ mainly due to unspecificity, and consequent uncontrolled side effects since a minimal level of ROS is needed for normal functioning.

Recently, cerium oxide nanoparticles (CeO_2_NPs) have emerged as a new powerful antioxidant agent with therapeutic properties in experimental liver disease. CeO_2_NPs have been reported to act as a ROS and NOS scavengers^22^ and to have multi-enzyme mimetic activity, including SOD activity^23^ (disproportionation of superoxide anion into oxygen and hydrogen peroxide), catalase activity^24^ (conversion of hydrogen peroxide into oxygen and water) and peroxidase activity^25^ (reducing hydrogen peroxide into hydroxil radicals). Consequently, the beneficial effects of CeO_2_NPs treatment have been reported in many different medical fields such as neurology^26^, ophthalmology^27^, cardiology^28^, oncology^29^ and hepatology^30^, among others. Unlike other antioxidants, CeO_2_NPs are only active at pathogenic levels of ROS, being inert and innocuous in healthy cells^31^. In this regard, previous investigations indicated that CeO_2_NPs are able to reduce steatosis^30^, attenuate oxidative stress^32–34^ and display anti-inflammatory properties^30,35,36^ in different experimental models of liver disease. Hence, we hypothesize that CeO_2_NPs could also have the potential to reduce steatosis, oxidative stress and inflammation in experimental MCDD-induced NAFLD. MCDD is widely used and one of the best characterized animal models to study NASH. Although it does not resemble the metabolic profile and etiology of human NAFLD, it mimics several of the histopathological features of human NAFLD^37,38^.

In the current study we explored the impact of CeO_2_NPs on steatosis by assessing the liver histology and fatty acid content, macrophage infiltration and the expression of genes involved in inflammation, ROS and lipid metabolism in the MCDD experimental model of NAFLD in rats. The aim of the investigation was to elucidate whether CeO_2_NPs reduce the accumulation of fat in the liver, oxidative stress, hepatic content of fatty acids and activation of proinflammatory genes in rats with MCDD-induced NAFLD.

## Results

### Characterization of CeO_2_NPs

**Figure 1:**
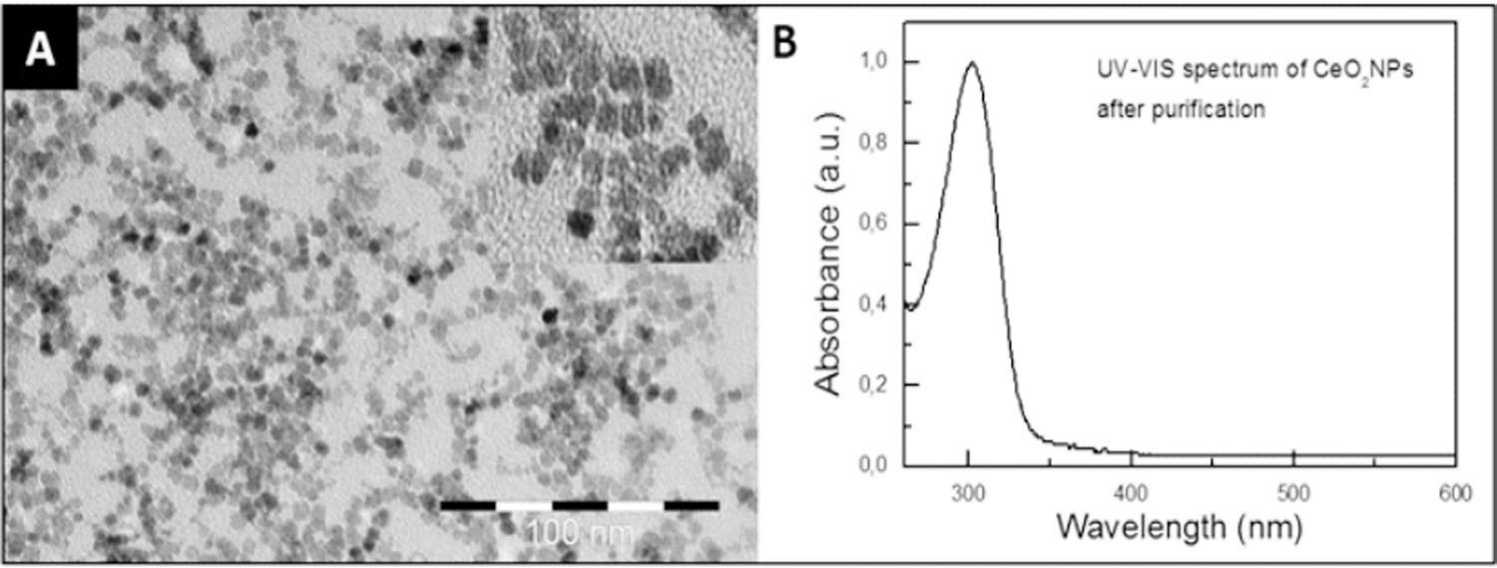
Characterization of the 4 nm CeO2NPs: (**A**) TEM image revealing CeO_2_NPs (scale bar is 100 nm) and (**B**) typical UV-VIS spectrum of the CeO_2_NPs after purification and resuspension in TMAOH 1 mM.

Details regarding the characterization of the CeO_2_NPs used in this article have been described in previous studies^30,39^. Briefly, HR-TEM analysis at high magnification suggested that the nanoparticles had a spherical morphology and were ~4 nm in diameter (Fig. 1). HR-TEM at low magnification revealed loose CeO_2_NPs agglomerates, and the X-ray diffraction pattern of CeO_2_NPs showed pure nanoparticles with the typical peak broadening characteristic of nanosize particles (data not shown).

### Body weight, liver to body weight ratio and serum biochemical parameters in control and MCDD rats treated with vehicle or CeO_2_NPs

In parallel to results previously obtained by other groups^38,40^, MCDD animals showed significantly decreased body weight and increased liver to body weight ratio than control rats (Table 1). Moreover, they also showed a remarkable alteration in plasma biomarkers of liver function. As shown in Table 1, rats fed with the MCDD displayed increased activity of transaminases, hypocholesterolemia, hyperbilirubinemia and significantly decreased levels of circulatory triglycerides. However, we were unable to detect any significant difference between rats treated and non-treated with CeO_2_NPs in any of the parameters assessed.

**Table 1:** Body weight, liver body weight ratio, and serum biochemical parameters test results in control and MCDD rats treated with vehicle or CeO_2_NPs.

	Control (n = 5)	MCDD rats	
		Vehicle (n = 8)	CeO_2_NPs (n = 7)
Body weight (g)	425.6 ± 6.7	275.3 ± 4.8***	274.5 ± 3.4***
Liver/body weight (%)	2.9 ± 0.18	4.1 ± 0.17**	4.2 ± 0.17***
Alanine transaminase (U/L)	35.3 ± 3.1	179.1 ± 26.6**	199.8 ± 24.2***
Aspartate transaminase (U/L)	49.1 ± 6.4	143.8 ± 14.52*	149.1 ± 15.1**
Gamma Glutamyl Transpeptidase (U/L)	2.1 ± 0.02	3.2 ± 0.5	2.8 ± 0.15
Total cholesterol (mg/dL)	69.4 ± 4.9	25.8 ± 1.7***	28.4 ± 2***
Total bilirubin (mg/dL)	0.00 ± 0.08	0.29 ± 0.06*	0.24 ± 0.05
Triglycerides (mg/dL)	192.2 ± 14.8	20.2 ± 1.4***	22.7 ± 2.1***
Glucose (mg/dL)	145.1 ± 6.9	120.2 ± 6.9	126.6 ± 4.6
Total proteins (g/L)	65.8 ± 1.2	63.3 ± 1.4	63.1 ± 1.2
Albumin (g/L)	34.9 ± 0.5	37.1 ± 0.7	37.2 ± 0.64

*p < 0.05,**p < 0.01, ***p < 0.001 compared to control group. One-way ANOVA with Newman-Keuls post hoc test and Kruskal-Wallis with Dunn’s test post hoc when appropriate. Results are shown as mean ± SEM.

### Histological examination of steatosis, inflammation and fibrosis in liver tissue

**Figure 2:**
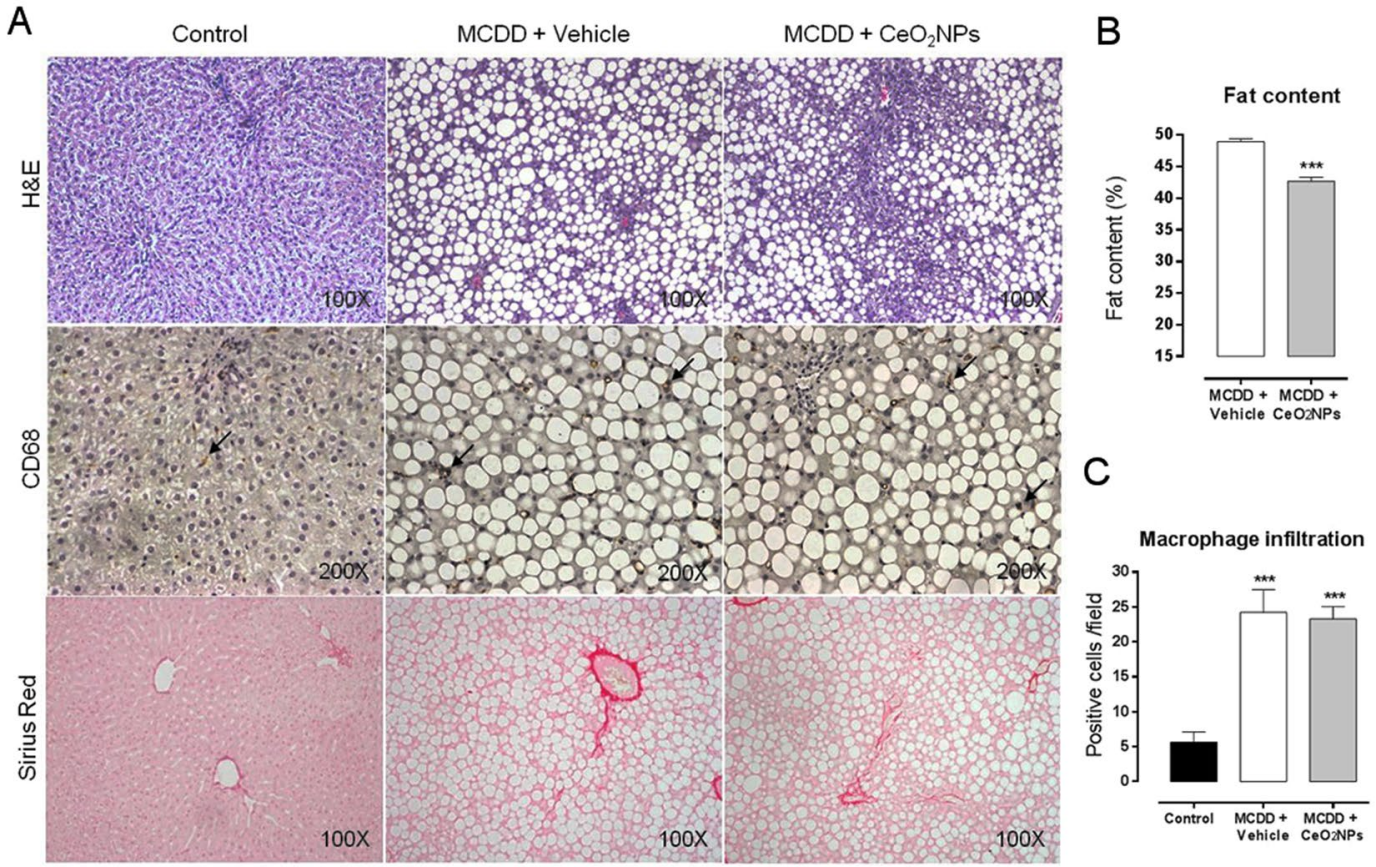
Effect of CeO_2_NPs on hepatic histology. (**A**) Hematoxylin & Eosin, CD68 and Sirius Red staining of representative liver sections obtained from control rats, MCDD rats receiving vehicle and MCDD rats receiving CeO_2_NPs. Original magnification 100x for H&E and Sirius Red, and 200x for CD68. (**B**) Quantitative measurement of fat content (%) in MCDD animals. ***p < 0.001 compared to vehicle group. Unpaired Student’s *t* test. (**C**) Quantitative measurement of CD68 positive cells/field in all animals. ***p < 0.01 compared to control group. One way ANOVA with Turkey’s multiple comparison test. Results are shown as mean ± SEM.

Figure 2A illustrates representative images of H&E, CD68 and Sirius red staining in liver biopsies of control and MCDD rats receiving vehicle or CeO_2_NPs. Macrovesicular steatosis was observed in both groups of MCDD rats as single large fat intra cytoplasmatic droplets displacing the nucleus. This alteration, consistent with a well-defined histological pattern of NAFLD, was significantly less pronounced in MCDD rats receiving CeO_2_NPs. Actually, the morphometric measurement of fat revealed a significant decrease of both, lipid content (48,91 ± 3,61 vs. 42,67 ± 5,75; %p < 0.001) and fat size (69 ± 6 µm^2^ vs. 63 ± 9 µm^2^, p < 0.001) (data not shown) in rats receiving CeO_2_NPs compared to those receiving vehicle (Fig. 2B). In addition, the MCDD also resulted in a significant inflammatory cell infiltrate in the liver tissue. However, quantification of CD68-positive stained cells did not show statistical differences between MCDD rats treated or non-treated with CeO_2_NPs (Fig. 2C). Finally, MCDD rats showed mild perivenular and portal fibrosis, with no significant differences between rats receiving and non-receiving CeO_2_NPs.

### Hepatic lipid peroxidation

**Figure 3:**
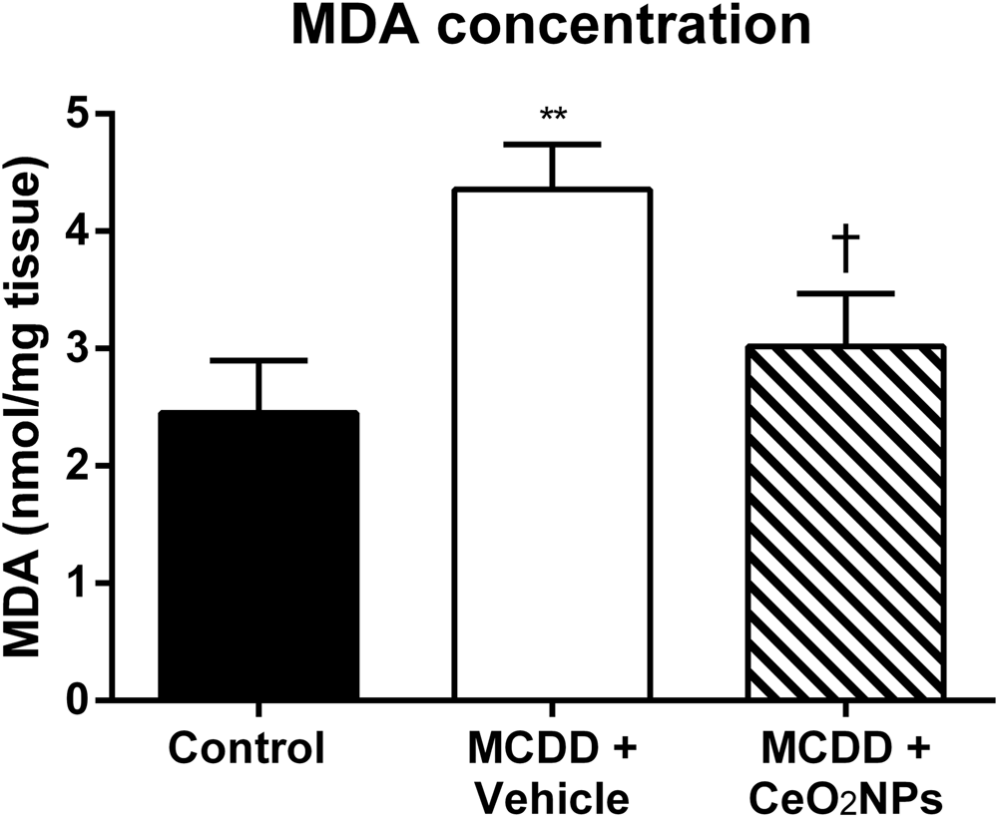
Levels of malondialdehyde (MDA) in MCDD fed rats. Content of MDA in the liver of control and MCDD non-treated (VH) and treated (CeO_2_NPs) rats (nmol/mg tissue). **p < 0.01 vs. control rats; ^†^p < 0.05 vs. MCDD rats receiving. VH Unpaired Student’s t-test. Results are given as means ± SEM.

In order to evaluate the oxidative stress-induced damage in the MCDD model of NAFLD and the antioxidant effects of CeO_2_NPs, lipid peroxidation was assessed by measuring malondialdehyde (MDA) content in the liver. A marked increment in the hepatic levels of MDA was found in MCDD rats treated with vehicle as compared to control rats. The level of MDA in the liver of the MCDD rats treated with CeO_2_NPs was significant lower than in those animals receiving vehicle (Fig. 3).

### Hepatic lipid profiling

Further information on the metabolic alterations associated with the diet-induced experimental NALFD model was obtained by measuring the principal lipid components in the liver of control and MCDD rats. As shown in Table 2, total FAs in TG, CE, PC and PE showed marked differences between control and MCDD rats. As anticipated, the liver content of total TG- and CE-derived FAs was markedly increased in MCDD rats in comparison to control animals. However, these differences were not seen on analyzing PC- and PE-derived FAs. On the contrary, in these cases we observed significantly reduced content of total FAs in the liver of MCDD animals. This can be explained by the lack of methionine and choline in the diet of the MCDD group. The effect induced by CeO_2_NPs administration on PC- and PE-derived FAs are shown in Tables 3 and 4 respectively.

**Table 2:** Total FA of principal lipid components in the hepatic tissue of control and MCDD rats (nmol/mg tissue).

	Control (n = 5)	MCDD (n = 8)
Triglycerides	199.8 ± 84.3	1348.9 ± 167.3***
Cholesterol Esters	6.7 ± 1.27	73.2 ± 9.4***
Phosphatidylcholines	1647.3 ± 90.4	825.8 ± 38.8***
Phosphatidylethanolamines	78.7 ± 7.3	60.8 ± 4.2*

Values are expressed as mean ± SEM. ***p < 0.001 and *p < 0.05, vs. control rats. Unpaired Student’s t-test.

**Table 3:** Content of PC-derived FAs in the liver of control and MCDD non-treated (vehicle) and treated (CeO_2_NPs) rats (nmol/mg tissue).

Fatty acid	Control (n = 4)	MCDD rats	
		Vehicle (n = 8)	CeO_2_NPs (n = 7)
C4:0	ND	ND	ND
C6:0	ND	ND	ND
C8:0	ND	ND	ND
C10:0	0.74 ± 0.14	1.46 ± 0.40	0.98 ± 0.19
C11:0	ND	ND	ND
C12:0	0.95 ± 0.19	1.25 ± 0.28	1.19 ± 0.16
C13:0	0.07 ± 0.01	0.18 ± 0.05	0.12 ± 0.03
C14:0	8.24 ± 0.94	2.84 ± 0.19***	2.53 ± 0.12**
C14:1	0.29 ± 0.04	0.35 ± 0.05	0.43 ± 0.05
C15:0	4.53 ± 0.14	1.93 ± 0.12***	1.68 ± 0.09**
C15:1	ND	ND	ND
C16:0	439.12 ± 39.28	237.78 ± 9.44***	236.56 ± 10.72**
C16:1	26.80 ± 8.10	3.54 ± 0.35**	3.43 ± 0.20**
C17:0	7.07 ± 0.97	2.74 ± 0.15***	2.71 ± 0.14**
C17:1	ND	ND	ND
C18:0	235.17 ± 29.60	147.84 ± 5.70**	149.53 ± 7.06**
C18:1n9	120.75 ± 16.74	54.88 ± 5.42***	52.05 ± 2.87**
C18:2n6	272.01 ± 23.96	119.42 ± 7.47***	116.67 ± 4.89**
C18:3n6	3.38 ± 0.50	2.82 ± 0.34	2.72 ± 0.26
C18:3n3	3.76 ± 0.25	3.02 ± 0.38	2.97 ± 0.35
C19:0	IS	IS	IS
C20:0	1.13 ± 0.04	0.77 ± 0.02***	0.83 ± 0.04**
C20:1n9	0.79 ± 0.09	0.49 ± 0.052**	0.55 ± 0.05*
C20:2	6.83 ± 0.69	1.55 ± 0.21***	2.17 ± 0.11**^,†^
c20:3n3	ND	ND	ND
C20:3n6	12.61 ± 1.91	6.24 ± 0.44**	6.63 ± 0.51*
C20:4n6	437.89 ± 16.47	201.99 ± 10.54***	226.46 ± 9.89**
C20:5n3	ND	ND	ND
C21:0	0.12 ± 0.01	0.11 ± 0.01	0.12 ± 0.01
C22:0	1.84 ± 0.14	1.59 ± 0.08	1.51 ± 0.11
C22:1n9	0.56 ± 0.12	0.63 ± 0.13	0.680 ± 0.13
C22:2	0.38 ± 0.08	0.51 ± 0.07	0.66 ± 0.04*
C22:6n3	51.68 ± 2.731	21.66 ± 2.11***	23.78 ± 1.62*
C23:0	1.59 ± 0.17	1.10 ± 0.10*	1.10 ± 0.09*
C24:0	6.58 ± 0.81	6.30 ± 0.47	6.41 ± 0.68
C24:1n9	2.49 ± 0.29	2.82 ± 0.28	3.50 ± 0.42
SFA	707.17 ± 54.23	405.88 ± 15.12**	405.26 ± 16.12**
UFA	940.22 ± 40.09	419.92 ± 24.39***	442.70 ± 17.16**
MUFA	151.67 ± 24.37	62.70 ± 5.65**	60.63 ± 2.84**
PUFA	788.54 ± 38.40	357.22 ± 19.72***	382.06 ± 14.57**

Values are expressed as mean ± SEM. SFA, saturated fatty acids; UFA, unsaturated fatty acids; MUFA, monounsaturated fatty acids; PUFA, polyunsaturated fatty acids; ND, non-detected peak; IS, internal standard. *p < 0.05, **p < 0.01, ***p < 0.001 vs. control rats; ^†^p < 0.05 vs. MCDD rats receiving vehicle. Unpaired Student’s t-test and Mann Whitney test when appropriate.

**Table 4:** Content of PE-derived FAs in the liver of control and MCDD non-treated (vehicle) and treated (CeO_2_NPs) rats (nmol/mg tissue).

Fatty acid	Control (n = 4)	MCDD rats	
		Vehicle (n = 8)	CeO_2_NPs (n = 7)
C10:0	ND	ND	ND
C11:0	ND	ND	ND
C12:0	ND	ND	ND
C13:0	ND	ND	ND
C14:0	0.09 ± 0.02	0.09 ± 0.03	0.04 ± 0.00*
C14:1	ND	ND	ND
C15:0	IS	IS	IS
C15:1	ND	ND	ND
C16:0	17.57 ± 1.68	13.24 ± 0.95*	12.67 ± 1.07*
C16:1	0.71 ± 0.18	0.22 ± 0.04**	0.21 ± 0.03**
C17:0	0.40 ± 0.06	0.26 ± 0.018*	0.26 ± 0.01*
C17:1	0.10 ± 0.02	0.10 ± 0.01	0.10 ± 0.01
C18:0	11.31 ± 2.29	13.19 ± 1.18	13.31 ± 1.00
C18:1n9	6.37 ± 0.88	4.42 ± 0.47	4.13 ± 0.35*
C18:2n6	8.21 ± 1.07	4.96 ± 0.53*	5.06 ± 0.42**
C18:3n6	0.16 ± 0.031	0.18 ± 0.03	0.17 ± 0.02
C18:3n3	0.38 ± 0.08	0.37 ± 0.09	0.39 ± 0.07
C19:0	ND	ND	ND
C20:0	0.06 ± 0.01	0.07 ± 0.01	0.06 ± 0.01
C20:1n9	ND	ND	ND
C20:2	0.20 ± 0.03	0.14 ± 0.02	0.16 ± 0.02
C20:3n3	ND	ND	ND
C20:3n6	0.69 ± 0.11	0.57 ± 0.04	0.61 ± 0.04
C20:4n6	27.55 ± 2.31	15.75 ± 1.11***	16.28 ± 1.00***
C20:5n3	ND	ND	ND
C21:0	ND	ND	ND
C22:0	0.05 ± 0.01	0.04 ± 0.01	0.05 ± 0.00
C22:1n9	ND	ND	ND
C22:2	ND	ND	ND
C22:6n3	4.81 ± 0.67	12.09 ± 1.25	7.10 ± 0.97***^,††^
C23:0	ND	ND	ND
C24:0	ND	ND	ND
C24:1n9	0.083 ± 0.026	0.081 ± 0.032	0.12 ± 0.02
SFA	29.48 ± 3.94	26.89 ± 2.16	26.39 ± 2.07
UFA	49.27 ± 3.48	33.89 ± 2.28**	39.32 ± 2.16*
MUFA	72.65 ± 1.01	4.82 ± 0.53*	4.55 ± 0.36*
PUFA	42.00 ± 3.31	29.07 ± 2.09**	34.76 ± 1.86

Values are expressed as mean ± SEM. SFA, saturated fatty acids; UFA, unsaturated fatty acids; MUFA, monounsaturated fatty acids; PUFA, polyunsaturated fatty acids. ND, non-detected peak; IS, internal standard. *p < 0.05, **p < 0.01, ***p < 0.001 vs. control rats; ^††^p < 0.01 vs. MCDD rats receiving vehicle. Unpaired Student’s t-test and Mann Whitney test when appropriate.

**Figure 4:**
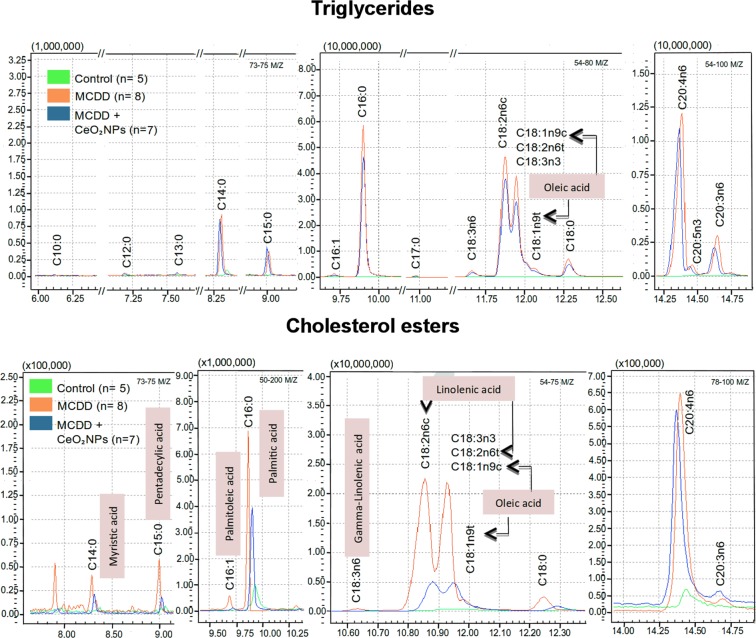
Liver fatty acid composition in control and MCDD rats. Superposition of representative gas chromatography mass spectrometry (GC/MS) chromatograms obtained from analysis of the TG- and CE-derived FAs of control (green) and non-treated (orange) and treated (blue) MCDD rats. GC peaks are labeled with the corresponding FA identification.

Marked abnormalities were found in both chromatographic patterns of TG- and CE-derived FAs of MCDD rats (Fig. 4). As compared to control animals, the most remarkable differences were, in TG-derived FAs, the presence of high or very high hepatic content of C16:0, C17:0, C18:0, C18:1n9, C18:2n6, C18:3n6, C18:3n3, C20:0, C20:1n9, C20:2, C20:3n6, C20:4n6, C20:5n3, C22:1n9 and C22:6n3 FAs (Table 5). This was due to a significantly increase of both saturated (SFA) and unsaturated (UFA) FAs. Furthermore, in the latter case this augmentation was a consequence of higher levels of both mono (MUFA) and poly UFA (PUFA). Moreover, the peroxidisability index (PI), an indicator of PUFA peroxidation^41–43^ that represents the degree of unsaturation of dietary lipids, was significantly higher in MCDD rats than in control animals (0.95 ± 0.05 vs 0.61 ± 0.06 nmol/mg tissue, p < 0.01). The pattern was quite similar in CE-derived FAs being C14:0, C15:0, C16:0, C16:1, C18:0, C18:1n9, C18:2n6, C18:3n6, C18:3n3, C20:0, C20:1n9, C20:2, C20:3n6, C20:4n6, C22:0 and C22:6n3 the FAs increased in this case. SFA and UFA were also found significantly increased in MCDD in comparison to control rats (Table 6). The PI was higher too, although did not reach statistical significance (1.08 ± 0.14 vs 0.67 ± 0.01 nmol/mg tissue).

**Table 5:** Content of Triglyceride-derived FAs in the liver of control, non-treated (vehicle) and treated (CeO_2_ NPs) MCDD rats (nmol/mg tissue).

Fatty acid	Control rats (n = 4)	MCDD rats	
		Vehicle (n = 8)	CeO_2_ NPs (n = 7)
C10:0	1.12 ± 0.71	0.52 ± 0.20	0.33 ± 0.09
C12:0	1.22 ± 0.64	0.48 ± 0.13	0.34 ± 0.07
C13:0	0.24 ± 0.1	0.04 ± 0.01	0.02 ± 0.0
C14:0	3.55 ± 1.99	5.32 ± 1.13	4.48 ± 0.57
C14:1	0.63 ± 0.36	0.20 ± 0.12	0.08 ± 0.02
C15:0	0.77 ± 0.36	1.94 ± 0.39	1.43 ± 0.16
C16:0	58.2 ± 24.5	303.4 ± 38.7***	220.5 ± 20.1***
C16:1	14.5 ± 9.4	8.3 ± 1.9	6.5 ± 0.8
C17:0	0.27 ± 0.12	1.73 ± 0.37*	1.35 ± 0.18 **
C17:1	0.46 ± 0.22	0.61 ± 0.17	0.43 ± 0.05
C18:0	3.08 ± 1.28	42.0 ± 5.9***	34.6 ± 4.1***
C18:1n9	44.3 ± 19.5	237.6 ± 28.6***	161.0 ± 17.8**^,†^
C18:2n6	54.8 ± 20.1	516.1 ± 66.6***	384.1 ± 47.2***
C18:3n6	1.2 ± 0.4	21.5 ± 4.3**	19.7 ± 2.5***
C18:3n3	4.0 ± 1.7	19.7 ± 3.1**	14.3 ± 2.**
C19:0	IS	IS	IS
C20:0	0.21 ± 0.11	0.61 ± 0.12*	0.43 ± 0.04
C20:1n9	0.51 ± 0.24	2.47 ± 0.53*	1.77 ± 0.19**
C20:2	0.34 ± 0.20	7.27 ± 1.66**	5.3 ± 0.65***
C20:3n6	1.28 ± 0.53	19.87 ± 4.81*	14.57 ± 1.71***
C20:4n6	5.2 ± 2.0	112. ± 18.8**	91.8 ± 9.3***
C20:5n3	1.2 ± 0.5	4.6 ± 0.7***	4.6 ± 0.6***
C21:0	0.08 ± 0.04	0.04 ± 0.00	0.03 ± 0.00
C22:0	0.19 ± 0.12	0.14 ± 0.02	0.09 ± 0.01
C22:1n9	0.10 ± 0.03	0.28 ± 0.05*	0.19 ± 0.03
C22:2	0.30 ± 0.16	0.49 ± 0.16	0.42 ± 0.07
C22:6n3	1.1 ± 0.5	14.5 ± 3.8*	12.9 ± 2.0**
C23:0	0.07 ± 0.03	0.031 ± 0.00	0.031 ± 0.00
C24:0	0.20 ± 0.15	0.08 ± 0.02	0.132 ± 0.0
C24:1n9	0.18 ± 0.05	0.19 ± 0.06	0.12 ± 0.02
SFA	69.3 ± 30.0	356.4 ± 45.4***	264.8 ± 24.0***
UFA	130.5 ± 54.3	966.6 ± 128.0***	718.0 ± 80.7***
MUFA	60.7 ± 29.6	249.8 ± 30.1**	170.1 ± 17.9**^,†^
PUFA	69.7 ± 25.2	716.8 ± 99.6***	547.9 ± 63.9***

Values are expressed as mean ± SEM. SFA, saturated fatty acids; UFA, unsaturated fatty acids; MUFA, monounsaturated fatty acids; PUFA, polyunsaturated fatty acids. ND, non-detected peak; IS, internal standard. *p < 0.05, **p < 0.01, ***p < 0.001 vs. control rats; ^†^p < 0.05 vs. MCDD rats receiving vehicle. Unpaired Student’s t-test and Mann Whitney test when appropriate.

**Table 6:** Content of CE-derived FAs in the liver of control and MCDD non-treated (vehicle) and treated (CeO_2_NPs) rats (pmol/mg tissue).

Fatty acid	Control (n = 4)	MCDD rats	
		Vehicle (n = 8)	CeO_2_NPs (n = 7)
C10:0	ND	ND	ND
C12:0	ND	ND	ND
C13:0	ND	ND	ND
C14:0	24.2 ± 6.0	250.3 ± 64.1*	60.8 ± 9.8*^,†^
C14:1	30.5 ± 6.7	38.4 ± 6.9	73.8 ± 12.9 *^,†^
C15:0	11.7 ± 6.6	117.5 ± 20.8**	52.5 ± 7.7**^,†^
C16:0	3634.4 ± 630.0	16464.0 ± 2863.0*	8204.5 ± 621.0**^,†^
C16:1	98.6 ± 69.5	625.7 ± 43.8***	503.1 ± 30.7***^,†^
C17:0	IS	IS	IS
C17:1	62.7 ± 16.1	64.4 ± 8.	96.371 ± 18.0
C18:0	347.8 ± 59.5	2503.4 ± 535.0*	1342.0 ± 169.0**
C18:1n9	711.9 ± 158.0	14427.7 ± 2298.0**	8602.7 ± 692.0***^,†^
C18:2n6	834.2 ± 151.0	26354.5 ± 3951.0**	16129.1 ± 1306.0***^,†^*^,†^
C18:3n6	43.1 ± 21.1	698.9 ± 153.0*	292.116 ± 17.2**^,†^
C18:3n3	77.5 ± 16.5	1127.9 ± 159.0**	828.6 ± 64.1***
C19:0	ND	ND	ND
C20:0	7.8 ± 2.4	59.8 ± 14.1*	38.0 ± 8.9*
C20:1n9	16.8 ± 7.6	159.6 ± 50*	46. ± 10.2*
C20:2	17.6 ± 6.5	277.6 ± 93.8**	87.4 ± 25.9**
C20:3n6	44.8 ± 12.5	653.4 ± 143.0*	411.600 ± 60.8**
C20:4n6	635.9 ± 114.0	8533.6 ± 1273.0**	9311.5 ± 831.0***
C20:5n3	ND	ND	ND
C21:0	5.4 ± 2.1	8.3 ± 0.7	12.6 ± 4.3
C22:0	7.3 ± 2.1	24.2 ± 3.3**	24.9 ± 5.2*
C22:1n9	49.2 ± 8.6	112.6 ± 27.1	133.6 ± 18.6*
C22:2	33.2 ± 11.7	69.3 ± 13.7	83.114 ± 17.6
C22:6n3	73.1 ± 21.4	557.8 ± 46.0***	997.4 ± 75.1***^,†††^
C23:0	5.3 ± 1.8	6.6 ± 1.1	9.4 ± 2.4
C24:0	9.1 ± 3.4	31.2 ± 5.3*	35.6 ± 7.1*
C24:1n9	ND	ND	ND
SFA	3966.4 ± 740.0	19465.8 ± 3471.0*	9780.7 ± 756.3***^,†^
UFA	2705.1 ± 520.1	53701.3 ± 6375.0***	37597.2 ± 2530.0***^,†^
MUFA	945.2 ± 228.5	15428.0 ± 2346.0**	9456.1 ± 697.8***^,†^
PUFA	1759.8 ± 323.2	38273.3 ± 4237.0***	28141.1 ± 2140.0***

Values are expressed as mean ± SEM. SFA, saturated fatty acids; UFA, unsaturated fatty acids; MUFA, monounsaturated fatty acids; PUFA, polyunsaturated fatty acids. ND, non-detected peak; IS, internal standard. *p < 0.05, **p < 0.01, ***p < 0.001 vs. control rats; ^†^p < 0.05, ^†††^p < 0.001 vs. MCDD rats receiving vehicle. Unpaired Student’s t-test and Mann Whitney test when appropriate.

**Figure 5:**
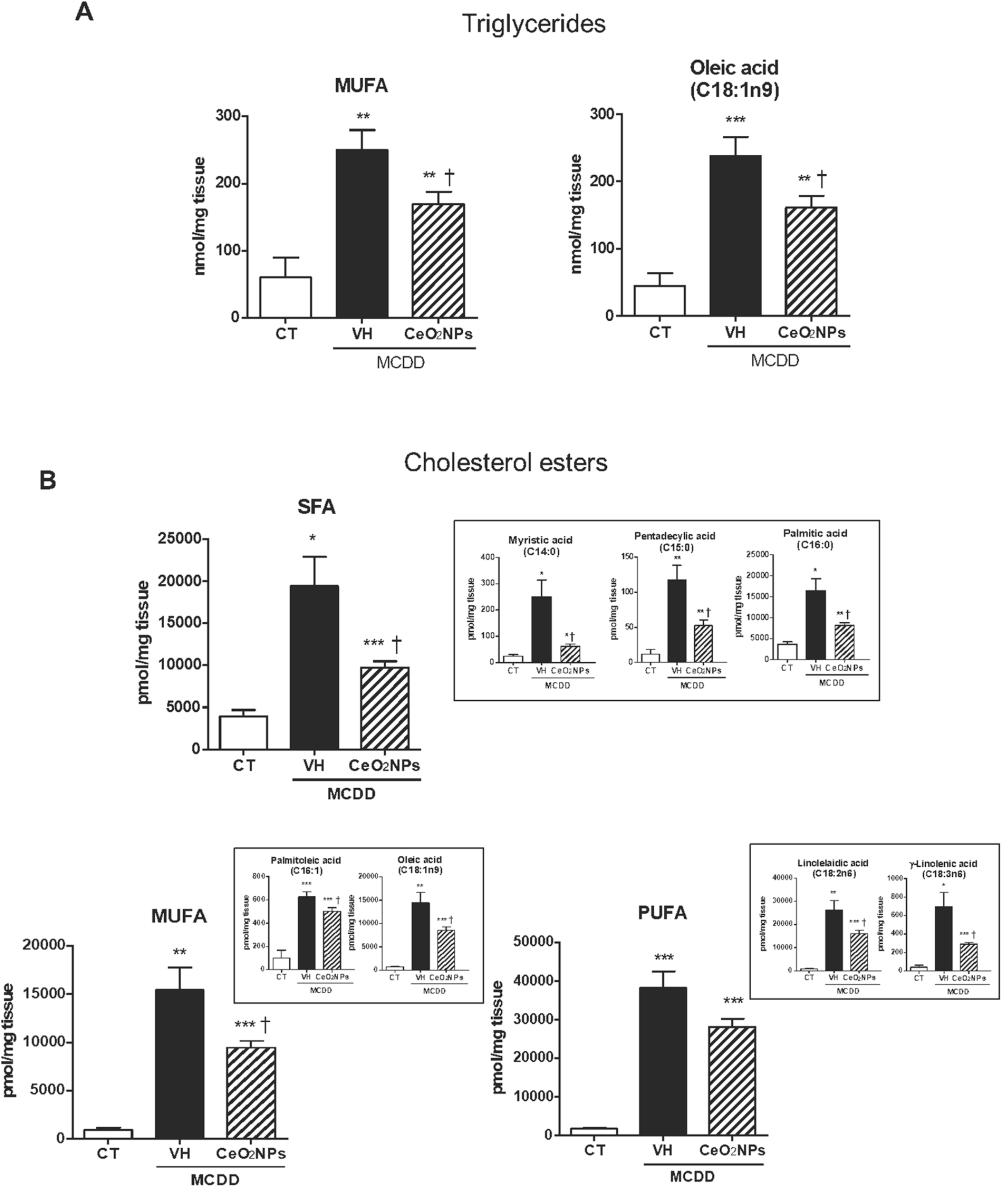
Effect of CeO_2_NPs on liver fatty acid composition in MCDD fed rats. (**A**) TG-derived FAs in the liver of control (CT) and MCDD non-treated (VH) and treated (CeO_2_NPs) rats (nmol/mg tissue). (**B**) Content of CE-derived FAs in the liver of CT and MCDD non-treated and treated rats (pmol/mg tissue). SFA, saturated fatty acids; MUFA, monounsaturated fatty acids; PUFA, polyunsaturated fatty acids. *p < 0.05, **p < 0.01, ***p < 0.001 vs. control rats; ^†^p < 0.05 vs. MCDD rats receiving VH Unpaired Student’s t-test. Results are given as means ± SEM.

Administration of CeO_2_NPs markedly altered the lipogenic activity in MCDD animals as indicated by a 26% and 33% decrease in the liver content of total TG and CE, respectively. A significant decrease in TG-derived MUFA, almost exclusively due to a diminution in TG-derived oleic acid, was observed (Fig. 5A). The most remarkable effects, however, were noted on analyzing CE-derived FAs. CeO_2_NPs treatment decreased SFA, MUFA and PUFA by approximately 50.7%, 38.7% and 25.6%, respectively (Fig. 5B). In the former case, this diminution was due to a lesser abundance of myristic, pentadecylic and palmitic acids, whereas palmitoleic and oleic acids and linolelaidic and γ-linolenic acids were the principal contributors in MUFA and PUFA, respectively. CeO_2_NPs did not significantly modify the altered PI in MCDD rats. Interestingly, we also observed that CeO_2_NPs induced a significant increase in the CE-derived very long chain PUFA, C22:6n3 (docosahexaenoic acid) (Table 6).

### Effect of CeO_2_NPs on fatty liver metabolism related gene expression in liver tissue

Further insight on the effect of CeO_2_NPs in the liver of MCDD rats was obtained by assessing messenger expression of 86 genes involved in fatty liver metabolism using a commercially available PCR array. Table 7 depicts all the genes showing a 2-fold or greater change in expression between the liver of MCDD rats receiving vehicle and that of control rats. Nine genes were significantly upregulated, including *Cd36*, a gene encoding for an enzyme involved in the adipokine signaling pathway, genes related to metabolic pathways (*Abcg1*, *Apoa1*, *Ctp1a*, *Gk* and *Lpl*), the *Il1β* gene, related to inflammatory response, and apoptosis-related genes (*Fas* and *Serpine1*). By contrast, four genes were significantly down-regulated, including those encoding insulin signaling pathway enzymes (*Igf1* and *Pklr*) or controlling other metabolic pathways (*Scd1* and *Slc27a5*).

**Table 7:** Messenger expression of genes involved in the pathogenic mechanisms of fatty liver showing 2-fold or greater regulation in liver between controls and MCDD rats treated with vehicle or CeO_2_NPs.

Genes	MCDD rats	
	Vehicle (n = 5)	CeO_2_NPs (n = 4)
***Insulin Signaling Pathway:***		
*Igf1*	−2.14*	−4.27*^,†^
*Igfbp1*	43.05	16.71
*Pklr*	−3.51**	−4.23**
*Ppargc1a*	8.13	4.97**
*Slc2a4*	−4.88	−7.75*
*Socs3*	2.59	1.26
*Srebf1*	−1.75	−3.27*
***Adipokine Signaling Pathway:***		
*Adipor1*	2.09	1.05
*Cd36*	7.25**	3.23***^,†^
*Lepr*	2.76	−1.02^†^
*Slc2a1*	2.69	1.19
***Metabolic Pathways:***		
*Acly*	−2.18	−2.82
*Abcg1*	5.66*	2.83*
*Acaca*	−2.17	−4.17**
*Acadl*	2.25	1.59
*Acsm3*	−1.61	−2.76
*Apoa1*	2.61*	1.86*
*Apoc3*	−1.92*	−2.83**
*Atp5c1*	2.14	1.21
*Cyp2e1*	2.98	1.70
*Cyp7a1*	2.50	2.18
*Cpt1a*	3.07*	1.42^†^
*Fabp3*	3.35	2.05
*Fasn*	−2.87	−3.46
*G6pc*	−1.24	−2.02
*G6pd*	3.32	1.90**
*Gck*	*2*.*09*	*3*.*30*
*Gk*	2.33*	1.44
*Hmgcr*	3.07	1.98
*Lpl*	16.11*	9.24**
*Mlxipl*	−1.64	−2.34
*Nr1h4*	−2.18	−2.46*
*Pck2*	6.25	4.35*
*Pdk4*	3.01	1.24
*Ppard*	2.96	1.69
*Scd1*	−45.15***	−27.08**
*Slc27a5*	−5.52**	−7.02*
*Srebf2*	2.42	1.23
***Inflammatory Response:***		
*Il1B*	3.48*	1.10^†^
*Tnf*	6.33	2.65
***Apoptosis:***		
*Casp3*	2.34	1.14
*Fas*	4.41*	2.29*
*Serpine1*	13.76*	7.26*

*Abcg1*, ATP-binding cassette, subfamily G (WHITE), member 1; *Acaca*, Acetyl-coenzyme A carboxylase alpha; *Acadl*, Acyl-CoA Dehydrogenase, Long Chain; *Acly*, ATP citrate lyase; *Acsm3*, Acyl-CoA synthetase medium-chain family member 3; *Adipor1*, Adiponectin receptor 1; *ApoA1*, apolipoprotein A-1; *Apoc3*, Apolipoprotein C-III; *Atp5c1*, ATP synthase subunit gamma, mitochondrial; *Casp3*, Caspase 3; *Cd36*, Cd36 molecule (thrombospondin receptor); *Cpt1a*, Carnitine palmitoyltransferase 1a, liver; *Cyp2e1*, Cytochrome P450, family 2, subfamily e, polypeptide 1; *Cyp7a1*, Cytochrome P450, family 7, subfamily a, polypeptide 1; *Fabp3*, Fatty acid binding protein 3, muscle and heart; *Fas*, Fas (TNF receptor superfamily, member 6); *Fasn*, Fatty acid synthase; *G6pc*, Glucose-6-phosphatase, catalytic subunit; *G6pd*, Glucose-6-phosphate dehydrogenase; *Gck*, Glucokinase; *Gk*, Glycerol kinase; *Hmgcr*, 3-hydroxy-3-methylglutaryl-Coenzyme A reductase; *Igf1*, Insulin-like growth factor 1; *Igfbp1*, Insulin-like growth factor binding protein 1; *Il1B*, Interleukin 1 beta; *Lepr*, leptin receptor; *Lpl*, Lipoprotein lipase; *Mlxipl*, MLX interacting protein-like; *Nr1h4*, Nuclear receptor subfamily 1, group H, member 4; *Pck2*, Phosphoenolpyruvate carboxykinase 2 (mitochondrial); *Pdk4*, Pyruvate dehydrogenase kinase, isozyme 4; *Pklr*, Pyruvate kinase, liver and RBC; *Ppard*, Peroxisome proliferator-activated receptor delta; *Ppargc1a*, Peroxisome proliferator-activated receptor gamma, coactivator 1 alpha; *Scd1*, Stearoyl-Coenzyme A desaturase 1; *Serpine1*, Serpin peptidase inhibitor, clade E (nexin, plasminogen activator inhibitor type 1), member 1; *Slc27a5*, Solute carrier family 27 (fatty acid transporter), member 5; *Slc2a1*, Solute carrier family 2 (facilitated glucose transporter), member 1; *Slc2a4*, Solute carrier family 2 (facilitated glucose transporter), member 4; *Socs3*, Suppressor of cytokine signaling 3; *Srebf1*, Sterol regulatory element binding transcription factor 1; *Srebf2*, Sterol regulatory element binding transcription factor 2; *Tnf*, Tumor necrosis factor (TNF superfamily, member 2). *p < 0.05, **p < 0.01, ***p < 0.001 vs. control rats. ^†^p < 0.05 vs. MCDD rats receiving vehicle. Unpaired Student’s t-test.

**Figure 6:**
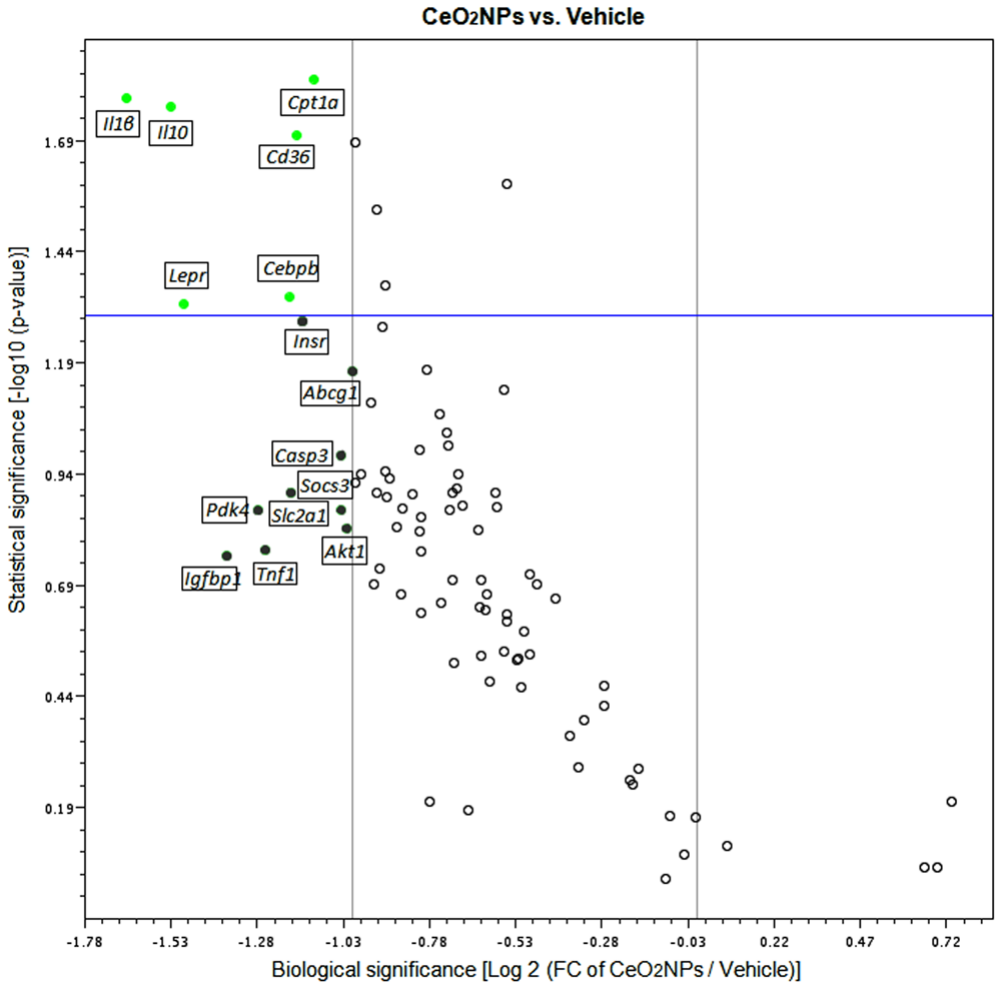
A volcano plot showing of the differentially expressed genes related to fatty liver in a pair-wise comparison of vehicle- and CeO_2_NP-treated MCDD rats. Significance was set at a p value based on a Student’s t-test of 0.05 [−log10 (p-value) ≥ 1.30]. The biological cut-off was set at a fold regulation of ±2 fold [−1 ≥ log2 (FC of CeO_2_NPs/Vehicle) ≥ 1]. The top 15 differentially expressed genes are labeled with their corresponding gene ID. The different color codes used represent biologically but not statistically significant genes (grey) and both biologically and statistically significant down-regulated (green) genes in CeO_2_NP treated rats.

A 2-fold or greater change in expression with p < 0.05 was considered statistically significant on comparing rats treated with vehicle vs. the CeO_2_NPs treated MCDD rats. Volcano plots of the data are presented in Fig. 6. Interestingly, CeO_2_NPs exerted a significant inhibitory effect on the expression of two genes related to the adipokine signaling pathway (*Cd36* and *Lepr*); one gene related to the fatty acid oxidation (*Cpt1a*) and the inflammatory response-related genes *Il1β*, *Il10* and *Cebpb*. A significant reduction in *Igf1* expression was also observed, but the biological significance of this data was roughly less than 2-fold.

### Effect of CeO_2_NPs on oxidative stress-associated gene expression in liver tissue

**Figure 7:**
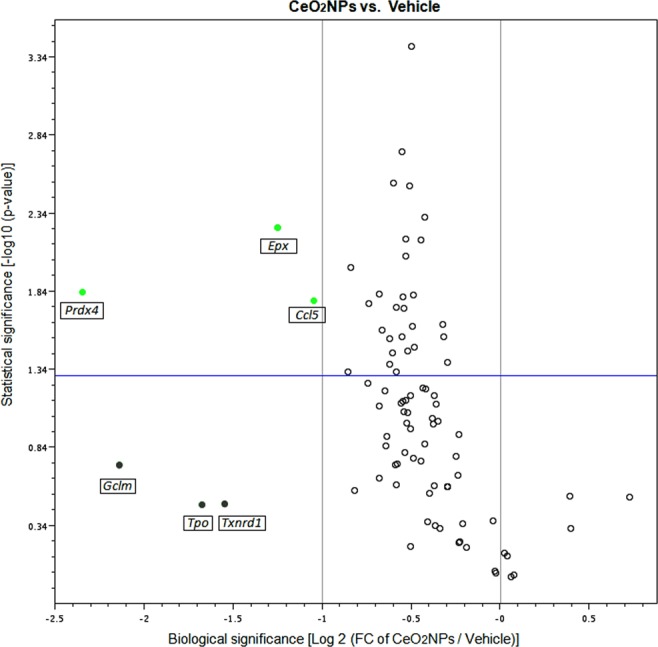
A volcano plot showing the differentially expressed genes related to oxidative stress in a pair-wise comparison of vehicle- and CeO_2_NP-treated MCDD rats. Significances was set at a p value based on a Student’s t-test of 0.05 [−log10 (p-value) ≥ 1.30]. The biological cut-off was set at a fold regulation of ±2 fold [−1 ≥ log2 (FC of CeO_2_NPs/Vehicle) ≥ 1]. The top 6 differentially expressed genes are labeled with their corresponding gene ID. The different color codes used represent biologically but not statistically significant genes (grey) and both biologically and statistically significant down-regulated (green) genes, in CeO_2_NP treated rats.

**Figure 8:**
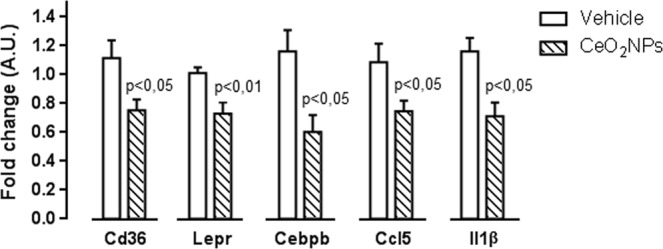
Effect of CeO_2_NPs on the expression of adipokine signaling, fatty acid oxidation and inflammation response-related genes in MCDD rats. The messenger RNA expression of *Cd36*, *Lepr*, *Cebpb*, *Ccl5* and *Il1β* was assessed by real-time PCR in liver tissue of MCDD rats treated with vehicle or CeO_2_NPs. Unpaired Student’s t-test. Results are given as mean + SEM.

The relative expression of 86 genes from several pathways involved in oxidative stress and antioxidant defense was assessed in the liver of MCDD rats treated with vehicle or CeO_2_NPs using a commercially available PCR array. Table 8 shows all the genes presenting a 2-fold or greater change in expression between the liver of the MCDD vehicle-treated group and that of control rats. MCDD induced significant changes in the expression of 31 genes; 27 were upregulated and 4 down regulated. The up-regulated genes included genes encoding enzymes involved in antioxidant metabolism (*Ehd2*, *Epx*, *Gpx2*, *Gpx3*, *Gpx7*, *Gstp1*, *Prdx2*, *Ptgs1*, *Ptgs2*, *Serpinb1b*, *Srxn1* and *Vimp*), genes related to ROS metabolism (*Ccl5*, *Cyba*, *Fmo2 Hmox1*, *Ncf1*, *Ncf2*, *Nos2*, *Prnp* and *Ucp2*), genes encoding oxygen transporters (*Cygb*, *Dnm2*, *Slc38a1* and *Vim*) and endoplasmic reticulum stress related genes (*Atf3* and *Ddit3*). Meanwhile, the four down-regulated genes, encoded the antioxidant enzyme catalase (*Cat*), genes controlling ROS metabolism (*Nox4* and *Scd1*) and one gene involved in oxygen transport (*Hba1*). A 2-fold or grater change in expression with p < 0.05 was considered statistically significant on comparing rats treated with vehicle vs. the CeO_2_NPs treated MCDD rats. Volcano plots of the data are presented in Fig. 7. CeO_2_NPs exerted a significant inhibitory effect on the expression of six genes related to antioxidant metabolism (*Epx*, *Gpx7*, *Gstp1*, *Prdx2*, *Prdx4* and *Vimp*) and four genes related to ROS metabolism (*Aox1*, *Ccl5*, *Hmox1* and *Ncf1*). However, an inhibitory effect greater than two fold was only observed on analyzing gene expression of *Epx*, *Prdx4* and *Ccl5*. To verify the results obtained by PCR array, we used quantitative RT-PCR to assess the expression level of most genes showing differential expression in the presence of CeO_2_NPs. The quantitative gene expression analysis demonstrated paralleled the results previously found in the array profiler. In fact, administration of CeO_2_NPs significantly decreased mRNA abundance of all the assessed genes (Fig. 8).

**Table 8:** Messenger expression of genes involved in oxidative stress and antioxidant defense showing 2-fold or greater regulation in liver between controls and MCDD rats treated with vehicle or CeO_2_NPs.

Genes	MCDD rats	
	Vehicle (n = 7)	CeO_2_NPs (n = 7)
***Antioxidants:***		
*Cat*	−2.86***	−2.92***
*Ehd2*	3.12**	2.41*
*Epx*	2.30*	−1.03^††^
*Gpx1*	−1.85**	−2.40***
*Gpx2*	68.00**	45.09**
*Gpx3*	12.53**	8.92**
*Gpx7*	8.16**	5.30***^,†^
*Gstp1*	3.63**	2.29**^,†^
*Mpo*	−7.82	−9.60
*Prdx2*	2.05**	1.46*^,†^
*Prdx4*	1.23	−4.13^†^
*Ptgs1*	3.03*	2.07**
*Ptgs2*	4.99*	3.51
*Serpinb1b*	11.73*	6.64
*Srxn1*	4.42**	3.07**
*Vimp*	2.85**	1.78**^,†^
***Reactive Oxygen Species (ROS) Metabolism:***		
*Aox1*	−1.94**	−3.52***^,†^
*Ccl5*	2.28*	1.10^†^
*Cyba*	6.57**	4.57**
*Fmo2*	2.93*	1.82
*Gclm*	1.22	−3.62
*Hmox1*	4.09**	2.65***^,†^
*Krt1*	2.27	1.72
*Ncf1*	5.19**	3.41**^,†^
*Ncf2*	5.25**	3.61*
*Nos2*	2,74*	2.75
*Nox4*	−2.64**	−2.01*
*Nqo1*	4.00	4.22*
*Prnp*	3.99*	2.55*
*Scd1*	−35.60***	−21.55***
*Tpo*	2.65	−1.20
*Txnrd1*	3.46	1.18
*Ucp2*	3.80**	2.79*
***Oxygen Transporters:***		
*Cygb*	3.29**	2.55
*Dnm2*	2.26*	1.53*
*Hba1*	−4.21**	−3.21*
*Slc38a1*	6.06**	3.62*
*Vim*	5.05**	4.30*
***ER stress***		
*Atf3*	27.35**	18.23*
*Ddit3*	2.76*	1.94**

*Aox*, Aldehyde oxidase 1; *Atf3*, Activating transcription factor 3; *Cat*, Catalase; *Ccl5*, C-C motif chemokine ligand 5; *Cyba*, Cytochrome b-245, alpha polypeptide; *Cygb*, Cytoglobin; *Ddit3*, DNA Damage Inducible Transcript 3; *Dnm2*, Dynamin 2; *Ehd2*, EH Domain Containing 2; *Epx*, Eosinophil Peroxidase; *Fmo2*, Flavin Containing Monooxygenase 2; *Gclm*, Glutamate cysteine ligase, modifier subunit; *Gpx1*, Glutathione peroxidase 1; *Gpx2*, Glutathione peroxidase 2; *Gpx3*, Glutathione peroxidase 3; *Gpx7*, Glutathione peroxidase 7; *Gstp1*, Glutathione S-transferase pi 1; *Hba1*, Hemoglobin alpha 1; *Hmox1*, Heme oxygenase (decycling) 1; *Krt1*, Keratin 1; *Mpo*, Myeloperoxidase; *Ncf1*, Neutrophil cytosolic factor 1; *Ncf2*, Neutrophil cytosolic factor 2; *Nos2*, Nitric oxide synthase 2, inducible; *Nox4*, NADPH oxidase 4; *Nqo1*, NAD(P)H dehydrogenase, quinone 1; *Prdx2*, Peroxiredoxin 2; *Prdx4*, Peroxiredoxin 4; *Prnp*, Prion protein; *Ptgs1*, Prostaglandin-endoperoxide synthase 1; *Ptgs2*, Prostaglandin-endoperoxide synthase 2; *Scd1*, Stearoyl-Coenzyme A desaturase 1; *Serpinb1b*, Serine (or cysteine) peptidase inhibitor, clade B, member 1b; *Slc38a1*, Solute carrier family 38, member 1; *Srxn1*, Sulfiredoxin 1 homolog; *Tpo*, Thyroid Peroxidase; *Txnrd1*, Thioredoxin Reductase 1; *Ucp2*, Uncoupling protein 2 (mitochondrial, proton carrier); *Vim*, Vimentin; *Vimp*, VCP-interacting membrane protein.

*p < 0.05, **p < 0.01, ***p < 0.001 vs. control rats; ^†^p < 0.05, ^††^p < 0.01 vs. MCDD rats receiving vehicle. Unpaired Student’s t-test.

## Discussion

In the current study we explored the effects of CeO_2_NPs on hepatic steatosis, inflammatory response, oxidative stress and liver fatty acid content in a MCDD-induced animal model of NAFLD. MCDD is among the most commonly used experimental methods to quickly induce liver steatosis and other hallmarks of NAFLD^44^. This diet, with high sucrose and 10% fat, but deficient in methionine and choline, results in macrovesicular steatosis within 3–4 weeks, progressing to inflammation and fibrosis^45^. As in previous investigations^38^, MCDD rats in the present study showed malnutrition, weight loss and a proportional increase in liver weight. Liver injury induced by MCDD was also associated with reduced serum levels of triglycerides and cholesterol, likely due to hepatic blockade of VLDL synthesis. Three weeks after starting the administration of the MCDD, when NAFLD was fully established but without reaching the most severe type of NASH, the iv administration of CeO_2_NPs was initiated. No noticeable effects on serum biochemistry parameters were observed following two weeks of CeO_2_NPs treatment. However, nanoparticles were able to reduce the hepatic fat content and the lipid droplet size in diet induced NAFLD animals. These apparently contradictory results at first may be explained by the fact that probably after 6 weeks of MCDD administration NAFLD intensity is already very high, and the significant steatosis reduction induced by CeO_2_NPs is not sufficient to represent a change in the analyzed serum parameters.

It is well known that feeding rats with MCDD increases lipid peroxidation and oxidative stress, which results in hepatocellular damage^45,46^. Actually, in the current investigation we observed a significant increment of MDA concentration in the liver of the MCDD rats, confirming thus the lipid peroxidation in these animals. Interestingly, CeO_2_NPs were able to reduce MDA concentration, indicating a marked reduction in the intensity of the lipid peroxidation. Moreover, this diet also results in remarkably increased hepatic FA accumulation^47^. Thus, we next sought to precisely assess whether CeO_2_NPs modify the FA pattern in animals with NAFLD. As expected, a dramatic difference in liver FA composition was observed between MCDD and control rats. MCDD animals showed between 2 to 30-fold higher FA content in hepatic TG and CE. Interestingly, regardless of which principal lipid component they were derived, the majority of these FAs were MUFA and PUFA. Interestingly, a marked reduction in SFAs and UFAs was observed in our MCDD animals treated with CeO_2_NPs. In view of the alterations induced by CeO_2_NPs, the goal of the next part of the study was to investigate the changes in expression of genes involved in hepatic lipid and ROS metabolism as a result of the MCDD, and to compare these changes to those obtained following CeO_2_NPs administration. The fatty liver and oxidative stress RT^2^ Profiler™ arrays used to determine hepatic gene expression have previously been successfully used in rat liver tissue^30,47,48^. In the present study we identified 14 MCDD-induced genes involved in β-oxidation pathways, adipokine signaling, inflammation and antioxidant and ROS metabolism that were significantly down-regulated or even normalized following CeO_2_NPs administration. One additional gene related to antioxidant metabolism that was not induced by the MCDD was also markedly down-regulated by the administration of CeO_2_NPs. Finally, we also identified two genes involved in insulin signaling and ROS metabolism that displayed decreased mRNA expression in MCDD fed rats, an effect further accentuated when the animals also received CeO_2_NPs. The molecular function of most of these genes, including Gstp1, Hmox1, Igf1, Il1β, Il10, Ccl5, Cebpb, Cd36 and Cpt1a was related to protein binding interaction. However, a more stringent analysis of the alterations in gene expression induced by CeO_2_NPs in MCDD fed animals, considering only those genes showing both statistical and biological significance, revealed two major groups of genes involved in lipid oxidation and cytokine signaling. The former group comprises Epx, Prdx4 and Cpt1a whereas the second group is formed by Il1β, Il10, Ccl5 (RANTES), Cd36, Lepr and Cebpb.

Analysis of the tissue FA profile has become increasingly important in understanding the role of lipids in physiological or pathological processes^49,50^. FAs are the essential components of lipids. In the recent years, it has been suggested that FAs play important roles as intracellular signaling molecules involved for instance in turning on nuclear receptors, including the peroxisome proliferator activate receptors (*Ppar*) which regulate lipid and carbohydrate metabolism transport and cellular proliferation^51–53^. In addition, it has been reported that a high presence of unsaturated FAs could lead to an increased of ROS production and cause mitochondrial permeability leading to apoptosis or necrosis^54^. In this regard, oxidative stress has been proposed as a major mechanism involved in NAFLD pathology. The antioxidant N-acetylcysteine has been found to attenuate the progression of NASH^55^. In the present study, the analysis of FAs hepatic composition showed a dramatic increase of FA in MCDD compared to control rats. Interestingly, the majority of these FAs were UFA (both MUFA and PUFA), which are more vulnerable to oxidation by free radicals and are pointed as a major source of lipid peroxidation^56^. Lipid peroxidation is a mechanism involved in the development and progression of NASH^57,58^ triggered specifically by the presence of ROS^59^. Oxidizing agents such as ROS can attack double bonded FAs, especially PUFAs, producing lipids with peroxide and hydroxyperoxide radicals^56^. Lipid peroxides can have lipotoxic effects on mitochondrial DNA, RNA and mitochondrial machinery proteins, thus contributing to mitochondrial dysfunction^60^. Among the secondary products of lipid peroxidation are MDA and 4-hydroxinonenal (HNE)^61^ that contributes to liver fibrosis and inflammation^62,63^. In this study we observed a marked increment of lipid peroxidation in the liver of the MCDD rats, and, furthermore, we demostrated that CeO_2_NPs have the potential to significantly reduce this process, thus, attenuating the associated lipotoxic effects. N-acetyl cysteine (NAC) is a widely used antioxidant, often used to protect the liver from oxidative damage caused by ROS. Due to the short half-life of NAC, it is useful to treat acute oxidative stress, however, it is not practical for the treatment of chronic oxidative stress^64,65^. On the other side, CeO_2_NPs remain deposited in the liver for a period of at least 30 days^66^. This property, together with their regenerative nature, would make the use of nanoceria a more interesting antioxidant approach against chronic oxidative stress^66^. Previous studies have compared the biological effects exerted by CeO_2_NPs with that exerted by NAC, demonstrating that CeO_2_NPs have similar antioxidant effects compared to NAC, but a longer half-life. Specifically, CeO_2_NPs showed a trend for greater inhibition of lipoperoxidation and ROS production compared to the NAC-treated animals in mice with liver damage^66^, a similar ability to increase GSH compared to NAC in cells under H_2_O_2_-induced oxidative stress^67^, and a stronger protective effect than NAC reducing ROS production and the apoptosis due to the TNF and cycloheximide administration in U937 cells^68^. Taken together, these studies suggest that CeO_2_NPs antioxidant effects are similar to the exerted by NAC, however, given their long-time deposition in the tissue and their regenerative capacity, CeO_2_NPs could exert a sustained antioxidant effect that may be more useful for chronic oxidative stress treatment.

Previous studies have shown significantly augmented ROS production by liver mitochondria in MCDD rodents. In particular, they exhibit excessive production of H_2_O_2_^69^. The marked reduction of both SFAs and UFAs observed in our MCDD animals treated with CeO_2_NPs is likely due to a diminished ROS production derived from the antioxidant properties of these nanoparticles. This would be also consistent with previous data from our laboratory showing that CeO_2_NPs reduce oxidative stress in H_2_O_2_-stimulated human derived cancer cells in culture^30^. In addition, CeO_2_NPs treatment also resulted in a significant increase in docosahexaenoic acid, a modulator of inflammatory response which has been considered as a potential treatment for NAFLD in children^70^. The current investigation provides for the first-time evidence that CeO_2_NPs induce changes in the hepatic gene expression of markers related to fatty liver and oxidative stress in the liver of MCDD fed rats, including *Epx*, *Prdx4*, *Cpt1a*, *Il1β*, *Il10*, *Ccl5* (RANTES), *Cd36*, *Lepr* and *Cebpb*. *Epx* and *Prdx4* are peroxidases whereas *Cpt1a* catalyzes the transfer of long chain FAs to carnitine for translocation across the mitochondrial inner membrane^71–73^. Moreover, increased *Cpt1a* expression has been previously observed in MCDD rats^74^. Our findings suggest that by decreasing *Cpt1a* expression CeO_2_NPs may improve the impairment in FA β-oxidation occurring in NAFLD animals. On the other hand, *Il1β* is one of the most potent proinflammatory cytokines and *Cd36* and *Lepr* are involved in adipokine signaling. *Cd36* mediates the cellular uptake of very long chain fatty acids and is known to be upregulated under hyperlipidemic conditions, contributing to the onset of hepatic steatosis^75^. Moreover, it has been shown that hepatocyte-specific disruption of *Cd36* in high-fat diet mice reduces liver CE and TC (the largest being oleic acid) and improves inflammatory markers^76^. *Lepr* is the receptor for leptin. *Ccl5* is a broader activator of several chemokine receptors, including *Ccr1*, *Ccr3*, *Ccr4* and *Ccr5* and it has been claimed as an important player in the pathophysiology of NAFLD-progression^77^. Finally, *Cebpb* is an important transcription factor that regulates the expression of genes involved in immune and inflammatory response which has been suggested to play a pivotal role in the pathogenesis of experimental NASH^78^. Taken together these results indicate that major beneficial effects of CeO_2_NPs are related to an anti-inflammatory effect secondary to general disruption of cytokine signaling.

In this study the experiments were performed using the MCDD experimental model of NAFLD. This model induces changes in the liver by nutritional deficiency and does not reflect the metabolic profile and the main etiopathogenic factors of human NAFLD^79–81^. However, in contrast to other NAFLD models, MCDD induces reproducible histological features of human NAFLD, with significant inflammation, fibrogenesis, and a liver redox balance similar to human patients^37,38^. Therefore, MCDD model is indicated for studying the consequences of fat accumulation, inflammation, oxidative stress and fibrogenesis in the liver^38^. Also, the MCDD model show sex differences regarding the degrees of steatosis and hepatic lipid content being higher in male rats than in female rats. The mechanisms underlying these differences are uncertain^82^. The current study has been performed in male Wistar rats, therefore, further studies would be necessary to confirm that the results are reproduced in females. Liver and spleen are the main target organs of CeO_2_NPs when administered intravenously^30,66,83^, thus, in this study, CeO_2_NPs were administered by iv route the rats to assure a high deposition in the liver. No toxicity or serious side effects were observed due to CeO_2_NPs iv administration, however, given the potential therapeutic value of CeO_2_NPs in NAFLD it would be interesting to explore less invasive routes of administration. In this respect, some studies indicate that oral administration of CeO_2_NPs show higher excretion of the nanoceria and less accumulative nanodeposition than intravenous and intraperitoneal administration^66,83^. Even so, liver remains as the main target for CeO_2_NPs deposition after oral administration^84^. Furthermore, anti-inflammatory properties of CeO_2_NPs have been proved when administered intragastrically, preventing and liver injury in an obesity experimental model^33^. Still, further studies are required to enhance nanoceria administration options.

In conclusion, the results of the current investigation show that in the MCDD experimental model of NAFLD characterized by malnutrition, weight loss, reduced serum levels of triglycerides and cholesterol, activation of liver tissue proinflammatory pathways, enhanced liver concentration of FAs and significant overexpression of genes related to fatty liver and ROS metabolism, the administration of CeO_2_NPs reduced the size and content of hepatocyte lipid droplets, the hepatic concentration of TG-derived MUFA, CE-derived SFA and UFA and messenger expression of several genes involved in cytokine, adipokine and chemokine signaling pathways. These findings, therefore, suggest that CeO_2_NPs could be of beneficial value in NALFD.

## Methods

### Synthesis and characterization of CeO_2_NPs

4 nm CeO_2_NPs were synthesized by the chemical precipitation of cerium (III) nitrate hexahydrated (Sigma-Aldrich, St. Louis, MO, USA) in a basic aqueous solution^24^. Briefly, 10 mM of cerium (III) nitrate hexahydrate was dissolved in 100 ml of milliQ H_2_O at RT. The solution was left for 30 minutes under stirring. 1 mL of TMAOH 1.0 (0.02 M in H_2_O) was added to the 100 mL solution at a final concentration of 10 mM, then, the mixture was left under stirring for 24 hours. Afterwards, the CeO_2_NPs were centrifugated and resuspended in 1 mM TMAOH aqueous solution, which act as a stabilizer. CeO_2_NPs were kept at 4 °C until the administration to animals. The surface charge of the CeO_2_NPs was analyzed in a Z-sizer (Malvern, Worcestershire, UK) while the size was evaluated by high-resolution (HR-TEM) in the Tecnai G2 F20 at 200 kV (FEI, Oregon, USA). The crystal structure was analyzed by HR-TEM (Tecnai 200 kV) and XRD (Xpert Pannalytical, MA, USA), and the light interaction by UV-VIS spectroscopy (Shimatzu, Kyoto, Japan). The size distribution was analyzed by Image J (National Institutes of Health, Bethesda, MD, USA).

### Animal procedures and dietary induction of liver steatosis in rats

The studies were performed in 20 male adult Wistar rats (Charles-River, Saint Aubin les Elseuf, France). Fifteen rats were fed with methionine and choline deficient diet (MCDD, TD 90262, Harlan Teklad). Five control rats were fed *ad libitum* with standard chow (Teklad global 14% protein rodent maintenance diet, Envigo). After 6 weeks of MCDD, rats were euthanized by isofluorane overdose. Livers obtained from each animal were immediately frozen in dry ice and stored at −80 °C for further analysis or fixed in 10% buffered formalin for hematoxylin and eosin (H&E) and immunostaining analysis. Serum samples were also obtained and kept at −20 °C until further analysis.

### CeO_2_NPs administration in MCDD rats

CeO_2_NPs or vehicle were diluted in saline solution and given as a bolus (500 µl) through the tail vein. CeO_2_NPs (0.1 mg/kg bw) or vehicle (saline solution containing 0.8 mM TMAOH ammonium salts) were injected twice a week for 2 consecutive weeks starting at the third week after liver steatosis induction.

### Morphometric measurement of steatosis and fibrosis

Liver sections (4 µm) were stained with H&E and digital images were obtained at a magnification of 100x with a microscope (Eclipse E600; Nikon, Tokio, Japan) and a digital camera (RT-Slider Spot; Diagnostic Instruments, Sterling Heights, MI, USA). For all the cases, the settings of the digital camera, microscope and software were the same. Ten digital images were taken for each slide. Image segmentation was made by selecting a few distinct fat droplets serving as reference. Area and roundness (R) were measured for each object. The formula (4 × π × area/perimeter^2^) was used to calculate R, which is equal to 1 for perfectly round objects and decreases toward 0 for more irregular objects. Filters were set to exclude exceedingly large objects (~3800 µm^2^) and objects with low roundness (R ≤ 0.35), which typically represented sinusoidal and vasuclar spaces or optically clear artifacts instead of fat droplets. All the indentified objects were manually inspected to ensure the quality. Fat content was defined as the percentage of total surface area occupied by fat droplets. The results were analyzed using imaging software (ImageJ, National Institutes of Health, Bethesda, MD, USA).

Fibrosis measurement was performed as described previously^85^. Briefly, using 0.1% Sirius red F3B (Sigma Aldrich, St. Louis, MO, USA) in saturated picric acid (Sigma-Aldrich, St. Louis, MO, USA). The relative fibrosis area was assessed by analyzing 32 fields of Sirius red-stained liver sections per animal. Each field was acquired as described above and the results were analyzed using imaging software (ImageJ, National Institutes of Health, Bethesda, MD, USA). To evaluate the relative fibrosis area, the collagen area measured was divided by the net field area and then multiplied by 100. Subtraction of the vascular luminal area from the total field area yielded the final calculation of the net fibrosis area. The amount of fibrosis measured in each animal was analyzed, and the average value was presented as a percentage^85^.

### Immunodetection of CD68

Liver sections from fibrotic rats underwent microwave antigen retrieval to unmask antigens hidden by cross-linkage occurring during tissue fixation. Endogenous peroxidase activity was blocked by hydrogen peroxide pretreatment for 10 min and with 5% goat serum for 45 min. The sections were then stained with mouse anti-CD68 (1:150; AbD Serotec, Oxford, UK, RRID:AB_2291300) and incubated for 1.5 h at RT. For antigen detection, the LSAB 2 System-HRP (Dako Denmark A/S) was used, and antigen visualization was achieved with streptavidin peroxidase and counterstained with hematoxylin. Immunostaining was performed without the first antibody for the negative controls. Macrophages (CD68-positive cells) in the middle and margin of the septa were assessed by counting 20 random fields per each section. The mean cell count for each sample was calculated^85^.

### Total concentration of malondialdehyde (MDA) in liver tissue

MDA concentration in the liver of control and MCDD rats non-treated (Vehicle) or treated with CeO_2_NPs were measured with the Lipid Peroxidation (MDA) Assay Kit (Abcam, Cambridge, United Kingdom) by following the manufacturer’s instructions. Briefly, liver homogenates were prepared using lysis solution and a Dounce homogenizer. The samples and the MDA standards were mixed with TBA solution and incubated at 95 °C for 1 hour. After a 10 minutes incubation on ice the samples and standard were read spectrophotometrically at 532 nm. The MDA concentration in the samples was determined by comparison with the MDA standard curve and presented as MDA nmol/mg of liver tissue.

### Hepatic lipid profiling by mass spectrometry analysis

Liver tissue (50 mg) was homogenized in chloroform: methanol (2:1, v/v, Scharlab, Barcelona, Spain, 372978 and PanReact AppliChem, Darmstadt, Germany, 1310911611, respectively) containing 0.005% butylated hydroxytoluene (Sigma Aldrich, Saint Louis, USA, W218405). Glyceryl trinonadecanoate (TG (19:0/19:0/19:0) Sigma Aldrich, Madrid, Spain), cholesteryl heptadecanoate (CE (17:0)), 1,2-dinonadecanoyl-sn-glycero-3-phosphocholine (PC (19:0/19:0)) and 1,2-dipentadecanoyl-sn-glycero-3-phosphoethanolamine (PE (15:0/15:0)) were used as internal standards. Separation of triglycerides (TG), cholesterol esters (CE), phosphatidylcholines (PC) and phosphatidylethanolamines (PE) from total lipid liver extracts dissolved in chloroform was performed by solid-phase extraction (SPE) using aminopropyl silica columns as described previously^86,87^. First, the CE and TG fractions were eluted with chloroform. Thereafter, PC were eluted with chloroform: methanol (3:2, v/v), and finally, PE were eluted with methanol. In order to isolate CE and TG, the first fraction was evaporated under nitrogen stream, dissolved in hexane (Merck Millipore, Darmstadt, Germany, 104374) and transferred to a fresh preconditioned aminopropyl silica column preconditioned with hexane. Then CE were eluted with hexane, and TG were eluted with hexane: chloroform: ethylacetate (100:5:5, v/v).

All solvent fractions containing isolated lipids were dried under nitrogen stream and transesterified (FAME) with 0.5 M NAOH and boron trifluoride (Sigma Aldrich, Saint Louis, USA, B1252) in methanol. GC–MS analyses of FAME were performed on a Shimadzu GCMS QP2010 Ultra instrument (Kyoto, Japan) as previously described^88^. Briefly, final extracts were injected in spitless mode (valve opened at 1 min) into the gas chromatograph interfaced with a mass selective detector. Chromatographic separation was achieved on a Sapines-5MS+ capillary column (30 m × 0.25 mm internal diameter × 0.25 μm film thickness) from Teknokroma (Barcelona, Spain) with helium as a carrier gas at a constant velocity of 50 cm/s. The temperature program was set to begin at 50 °C, maintained at this temperature for 3 min, elevated at 80 °C min^−1^ to 240 °C, then increased at 2 °C min^−1^ until 290 °C and finally maintained for 2 min at 290 °C. The ion source and transfer line temperatures were set at 250 °C and 280 °C, respectively. The mass detector was operated in scan mode. Identification of the FAME in the sample extracts was achieved by mass spectrum and GC retention time comparison with reference standards (Sigma). Results are expressed as nmol of FA/mg liver tissue.

### Oxidative stress and fatty liver gene expression PCR array in the liver of MCDD rats

Total RNA was extracted using an RNA extraction column kit (RNAeasy, Qiagen, Venlo, The Netherlands). To remove residual DNA, RNA preparations were treated with RNase-Free DNAse set (Qiagen). First strand cDNA was synthesized from 500 ng total of RNA using an RT^2^ first-strand kit (Qiagen), and PCR arrays were performed according to the manufacturer’s protocols (SABiosciences, Frederick, MD). Real-time PCR arrays were performed using the rat Oxidative Stress RT^2^ Profiler^TM^ PCR array, (SABiosciences) and the rat Fatty Liver RT^2^ Profiler PCR array according to the manufacturer’s protocol. These PCR arrays combine the quantitative performance of SYBR Green-based real-time PCR with the multiple gene profiling capabilities of microarrays to profile the expression of 86 key genes involved in oxidative stress or NAFLD. PCR array plates were processed in a Light Cycler 480 (Roche Diagnostics) using automated baseline and threshold cycle detection. Normalization of gene expression was performed using internal controls to determine the fold change in gene expression between the test and the control samples. The relative quantity of product was expressed as fold-induction of the target gene compared with the reference gene according to the formula 2^−ΔΔCT^. Data were interpreted using the SABiosciences web-based PCR array data analysis tool (http://pcrdataanalysis.sabiosciences.com/pcr/arrayanalysis.php).

### RT-PCR validation

To confirm the effect induced by CeO_2_NPs on gene expression unveiled by the RT^2^ profiler we used RT-PCR. As described previously^89^, total RNA was extracted from the middle liver lobe of control and fibrotic rats using a commercially available kit (RNAeasy; QIAGEN, Hilden, Germany). The RNA concentration was determined by spectrophotometric analysis (ND-100 spectrophotometer; Thermo Fischer Scientific, Waltham, MA, USA). One microgram of total RNA was reverse-transcribed using a cDNA synthesis kit (High-Capacity cDNA Reverse Transcription Kit; Applied Biosystems, Foster City, CA, USA). The specific primers and probes used for the different genes studied were designed to include intron spanning using the Universal Prove Library Assay Design Center through ProbeFinder version 2.5 software (Roche Diagnostics, Indianapolis, IN, USA; http://lifescience.roche.com/shop/es/mx/overviews/brand/universal-probe-library). The panel included the following: CCAAT/Enhancer Binding Protein Beta (Cebpb) (probe 70: left 5′-CTTCAGCCCCTACCTGGAG-3′ and right 5′-GAGGTCGGAAAGGAAGTCGT-3′); interleukin 1 Beta (Il1β) (probe 76: left 5′-CAGGAAGGCAGTGTCACTCA-3′ and right 5′-TCCCACGAGTCACAGAGGA-3′); cluster of differentiation 36 (Cd36) (probe 75: left 5′-GCGACATGATTAATGGCACA-3′ and right 5′-TGGACCTGCAAATGTCAGAG-3′); leptin receptor (Lepr) (probe 66: left 5′-AAAGCACCATTTCCACTTCAA-3′ and right 5′-GCAGAGATGTATCCGAGACGA-3′) and C-C Motif Chemokine Ligand 5 (Ccl5) (probe 16: left 5′-CTCACCGTCATCCTCGTTG-3′ and right 5′-GAGTGGTCTCCGAGCCATA-3′). Hypoxanthine-guanine phosphoribosyltransferase (Hprt) (probe 95: right 5′-GACCGGTTCTGTCATGTCG-3′ and left 5′-ACCTGGTTCATCATCACTAATCAC-3′) was used as the reference gene. Primers were designed according to rat sequences (GenBank codes NM_001301715.1, NM_031512.2, NM_031561.2, NM_012596.1, NM_031116.3 and NM_012583.2). Real-time quantitative polymerase chain reaction was analyzed in duplicate and performed with the LightCycler 480 (Roche Diagnostics). A 10-µl volume reaction of diluted 1:8 cDNA, 200 nM primer dilution, 100 nM prevalidated 9-mer probe (Universal ProbeLibrary) and FastStart TaqMan Probe Master (Roche Diagnostics) were used in each PCR. A fluorescence signal was captured during each of the 45 cycles (denaturizing for 10 s at 95 °C, annealing for 20 s at 60 °C, and extension for 1 s at 72 °C). Water was used as a negative control. Relative quantification was calculated using the comparative threshold cycle (Ct), which is inversely related to the abundance of mRNA transcripts in the initial sample. The mean Ct of duplicate measurements was used to calculate ΔCt as the difference in Ct for target and reference. The relative quantity of product was expressed as fold induction of the target gene compared with the reference gene according to the formula 2−ΔΔCT, where ΔΔCt represents ΔCt values normalized with the mean ΔCt of control samples.

### Measurements and statistical analysis

Biochemical standard parameters of liver function were measured in the BS-200E Chemistry Analyzer (Mindray Medical International Ltd, Shenzhen, China). Quantitative data were analyzed using GraphPad Prism 5 (GraphPad Software Inc., San Diego, CA), and statistical analysis of the results was performed by unpaired Student’s t test, one-way analysis of variance (ANOVA),The Newman-Keuls post hoc test and the Kruskal-Wallis test with the Dunn post hoc test when appropriate. Unpaired Student’s t-test was also performed when appropriate. Results are expressed as mean ± standard error of the mean (SEM) and considered significant p ≤ 0.05.

### Ethical approval

The experimental protocol was approved by the Investigation and Ethics Commitee of the Hospital Clínic Universitari. All applicable international, national and institutional guidelines for the care and use of animals were followed, and the study was performed according to the ethical standards of the Investigation and Ethics Committee of the Hospital Clinic Universitari.

### Compliance with ethical standards

All applicable international, national, and/or institutional guidelines for the care and use of animals were followed. All procedures performed in studies involving animals were approved by the Investigation and Ethics Committee of the Hospital Clinic Universitari, and all the experiments were performed in accordance with their ethical standards.

## Data Availability

The datasets generated during and/or analyzed during the current study are available from the corresponding author on reasonable request.

## References

[1] Levene AP, Goldin RD (2012). The epidemiology, pathogenesis and histopathology of fatty liver disease. Histopathology..

[2] Vernon G, Baranova A, Younossi ZM (2011). Systematic review: the epidemiology and natural history of non-alcoholic fatty liver disease and non-alcoholic steatohepatitis in adults. Aliment. Pharmacol. Ther..

[3] Ertle J (2011). Non-alcoholic fatty liver disease progress to hepatocellular carcinoma in the absence of apparent cirrosis. Int. J. Cancer..

[4] Adams LA (2009). The natural history of nonalcoholic fatty liver disease: a population based cohort study. Gastroenterology..

[5] Brunt EM, Neuschwander-Tetri BA, Oliver D, Wehmeier KR, Bacon BR (2004). Nonalcoholic steatohepatitis: histologic features and clinical correlations with 30 blinded biopsy specimens. Hum. Pathol..

[6] Matteoni CA (1999). Nonalcoholic fatty liver disease: a spectrum of clinical and pathological severity. Gastroenterology..

[7] Caldwell SH, Chang CY, Nakamoto RK, Krugner-Higby L (2004). Mitochondria in nonalcoholic fatty liver disease. Clin. Liver. Dis..

[8] Angulo P (2002). Nonalcoholic fatty liver disease. N. Engl. J. Med..

[9] Farrell GC (2003). Non-alcoholic steatohepatitis: what is it, and why is it important in the Asia-Pacific region?. J. Gastroenterol. Hepatol..

[10] Sunny NE, Bril F, Cusi K (2017). Mitochondrial Adaptation in Nonalcoholic Fatty Liver Disease: Novel Mechanisms and Treatment Strategies. Endocrinol. Metab..

[11] Marra F, Svegliati-Baroni G (2018). Lipotoxicity and the gut-liver axis in NASH pathogenesis. J. Hepatol..

[12] Malhi H, Bronk SF, Werneburg NW, Gores GJ (2006). Free fatty acids induce JNK-dependent hepatocyte lipoapoptosis. J. Biol. Chem..

[13] Weltman MD, Farrell GC, Liddle C (1996). Increased hepatocyte CYP2E1 expression in a rat nutritional model of hepatic steatosis with inflammation. Gastroenterology..

[14] Li Z, Berk M, McIntyre TM, Gores GJ, Feldstein AE (2008). The lysosomal-mitochondrial axis in free fatty acid-induced hepatic lipotoxicity. Hepatology..

[15] Day CP (2002). Pathogenesis of steatohepatitis. Best. Pract. Res. Clin. Gastroenterol..

[16] Cusi K (2009). Nonalcoholic fatty liver disease in type 2 diabetes mellitus. Curr. Opin. Endocrinol. Diabetes. Obes..

[17] Forrester SK, Kikuchi DS, Hernandes MS, Xu Q, Griendling KK (2018). Reactive oxygen species in metabolic and inflammatory signaling. Circ. Res..

[18] Bril, F. *et al*. Role of Vitamin E for Nonalcoholic Steatohepatitis in Patients With Type 2 Diabetes: A Randomized Controlled Trial. *Diabetes*. *Care*. Dc190167 (2019). 10.2337/dc19-0167 31332029

[19] Hernández-Guerra M (2006). Ascorbic acid improves the intrahepatic endotelial dysfunction of patients with cirrhosis and portal hypertension. Hepatology..

[20] Laviña B (2009). Superoxide dismutase gene transfer reduces portal pressure in CCl_4_ cirrhotic rats with portal hypertension. Gut..

[21] Firuzi O, Miri R, Tavakkoli M, Saso L (2011). Antioxidant therapy: current status and future prospects. Curr. Med. Chem..

[22] Dowding JM, Dosani T, Kumar A, Seal S, Self WT (2012). Cerium oxide nanoparticles scavenge nitric oxide radical (NO). Chem. Commun..

[23] Korsvik C, Patil S, Seal S, Self WT (2007). Superoxide dismutase mimetic properties exhibited by vacancy engineered ceria nanoparticles. Chem. Commun..

[24] Cafun JD, Kvashnina KO, Casals E, Puntes VF, Glatzel P (2013). Absence of Ce^3+^ sites in chemically active colloidal ceria nanoparticles. ACS. Nano..

[25] Heckert EG, Seal S, Self WT (2008). Fenton-Like Reaction Catalyzed by the Rare Earth Inner Transition Metal Cerium. Environ. Sci. Technol..

[26] Kim CK (2012). Ceria nanoparticles that can protect against ischemic stroke. Angew. Chem. Int. Ed. Engl..

[27] Chen J, Patil S, Seal S, McGinnis JF (2006). Rare earth nanoparticles prevent retinal degeneration induced by intracellular peroxides. Nat. Nanotechnol..

[28] Niu J, Azfer A, Rogers LM, Wang X, Kolattukudy PE (2007). Cardioprotective effects of cerium oxide nanoparticles in a transgenic murine model of cardiomyopathy. Cardiovasc. Res..

[29] Alili L (2011). Combined cytotoxic and anti-invasive properties of redox-active nanoparticles in tumor-stroma interactions. Biomaterials..

[30] Oró D (2016). Cerium oxide nanoparticles reduce steatosis, portal hypertension and display anti-inflammatory properties in rats with liver fibrosis. J. Hepatol..

[31] Oostingh GJ (2011). Problems and challenges in the development and validation of human cell-based assays to determine nanoparticle-induced immunomodulatory effects. Part. Fibre. Toxicol..

[32] Hashem RM, Rashd LA, Hashem KS, Soliman HM (2015). Cerium oxide nanoparticles alleviate oxidative stress and decreases Nrf-2/HO-1 in D-GALN/LPS induced hepatotoxicity. Biomed. Pharmacother..

[33] Kobyliak N (2017). Cerium dioxide nanoparticles possess anti-inflammatory properties in the conditions of the obesity-associated NAFLD in rats. Biomed. Pharmacother..

[34] Ibrahim HG, Attia N, Hashem FEZA, El Heneidy MAR (2018). Cerium oxide nanoparticles: In pursuit of liver protection against doxorubicin-induced injury in rats. Biomed. Pharmacother..

[35] Ribera J (2019). Functionalized cerium oxide nanoparticles mitigate the oxidative stress and pro-inflammatory activity associated to the portal vein endothelium of cirrhotic rats. PLoS One..

[36] Adebayo OA, Akinloye O, Adaramoye OA (2019). Cerium Oxide Nanoparticles Attenuate Oxidative Stress and Inflammation in the Liver of Diethylnitrosamine-Treated Mice. Biol. Trace. Elem. Res..

[37] Begriche K, Igoudjil A, Pessayre D, Fromenty B (2006). Mitochondrial dysfunction in NASH: causes, consequences and possible means to prevent it. Mitochondrion..

[38] Kucera O, Cervinkova Z (2014). Experimental models of non-alcoholic fatty liver disease in rats. World. J. Gastroenterol..

[39] Verstraelen S (2014). Gene expression profiles reveal distinct immunological responses of cobalt and cerium dioxide nanoparticles in two *in vitro* lung epithelial cell models. Toxicol. Lett..

[40] George J (2003). Lipid peroxidation, stellate cell activation and hepatic fibrogenesis in a rat model of chronic steatohepatitis. J. Hepatol..

[41] Kang JM, Shin MS, Park JN, Lee SS (2005). The effects of polyunsatured:satured fatty acids ratios and peroxidisability index values of dietary fats on serum lipid profiles and hepatic enzyme activities in rats. Br. J. Nutr..

[42] Nagyová A, Krajcovicová-Kudlácková M, Klvanová J (2001). LDL and HDL oxidation and fatty acid composition in vegetarians. Ann. Nutr. Metab..

[43] Saito M, Kubo K (2003). Relationship between tissue lipid peroxidation and peroxidizability index after alpha-linolenic, eicosapentaenoic, or docosahexaenoic acid intake in rats. Br. J. Nutr..

[44] Santhekadur PK, Kumar DP, Sanyal AJ (2018). Preclinical models of non-alcoholic fatty liver disease. J. Hepatol..

[45] Veteläinen R, Van Vliet A, Van Gulik TM (2007). Essential pathogenic and metabolic differences in steatosis induced by choline or methionine.choline deficient diets in a rat model. J. Gastroenterol. Hepatol..

[46] Serviddio G (2010). A silybin-phospholipid complex prevents mitochondrial dysfunction in a rodent model of nonalcoholic steatohepatitis. J. Pharmacol. Exp. Ther..

[47] Pan QR (2015). Resveratrol prevents hepatic steatosis and endoplasmic reticulum stress and regulates the expression of genes involved in lipid metabolism, insulin resistance, and inflammation in rats. Nutr. Res..

[48] Suzuki M (2014). Uncoupling protein-2 is an antioxidant that is upregulated in the enamel organ of fluoride-treated rats. Connect. Tissue. Res..

[49] Puri P (2007). A lipidomic analysis of nonalcoholic fatty liver disease. Hepatology..

[50] Serviddio G (2016). Effects of dietary fatty acids and cholesterol excess on liver injury: A lipidomic approach. Redox. Biol..

[51] Grimaldi PA (2001). Fatty acid regulation of gene expression. Curr. Opin. Clin. Nutr. Metab. Care..

[52] Hihi AK, Michalik L, Wahli W (2002). PPARs: transcriptional effectors of fatty acids and their derivatives. Cell. Mol. Life. Sci..

[53] Jump DB (2004). Fatty acid regulation of gene transcription. Crit. Rev. Clin. Lab. Sci..

[54] Rohrbach S (2009). Effects of dietary polyunsaturated fatty acids on mitochondria. Curr. Pharm. Des..

[55] Baumgardner JN (2008). N-acetylcysteine attenuates progression of liver pathology in a rat model of nonalcoholic steatohepatitis. J. Nutr..

[56] Yin H, Xu L, Porter NA (2011). Free radical lipid peroxidation: mechanisms and analysis. Chem. Rev..

[57] Zamara E (2004). 4-Hydroxynonenal as a selective pro-fibrogenic stimulus for activated human hepatic stellate cells. J. Hepatol..

[58] Albano E (2005). Immune response towards lipid peroxidation products as a predictor of progression of non-alcoholic fatty liver disease to advanced fibrosis. Gut..

[59] Letteron P, Fromenty B, Terris B, Degott C, Pessayre D (1996). Acute and chronic hepatic steatosis lead to *in vivo* lipid peroxidation in mice. J. Hepatol..

[60] Abdul-Ghani MA (2008). Deleterious action of FA metabolites on ATP synthesis: possible link between lipotoxicity, mitochondrial dysfunction, and insulin resistance. Am. J. Physiol. Endocrinol. Metab..

[61] Esterbauer H, Cheeseman KH (1990). Determination of aldehydic lipid peroxidation products: malonaldehyde and 4-hydroxynonenal. Methods. Enzymol..

[62] Dixon LJ, Flask CA, Papouchado BG, Feldstein AE, Nagy LE (2013). Caspase-1 as a central regulator of high fat diet-induced non-alcoholic steatohepatitis. PLoS One..

[63] Dou X (2012). Inhibition of NF-κB activation by 4-hydroxynonenal contributes to liver injury in a mouse model of alcoholic liver disease. Am. J. Pathol..

[64] Jackson IM, Barnes J, Cooksey P (1984). Efficacy and tolerability of oral acetylcysteine (Fabrol) in chronic bronchitis: a double-blind placebo controlled study. J. Int. Med. Res..

[65] Wu YJ, Muldoon LL, Neuwelt EA (2004). The chemoprotective agent N-acetylcysteine blocks cisplatin-induced apoptosis through caspase signaling pathway. J. Pharmacol. Exp. Ther..

[66] Hirst SM (2013). Biodistribution and *in vivo* antioxidant effects of cerium oxide nanoparticles in mice. Environ. Toxicol..

[67] Akhtar MJ, Ahamed M, Alhadlaq HA, Khan MAM, Alrokayan SA (2015). Glutathione replenishing potential of CeO_2_ nanoparticles in human breast and fibrosarcoma cells. J. Colloid. Interface. Sci..

[68] González-Flores D (2014). Nanoceria protects from alterations in oxidative metabolism and calcium overloads induced by TNFα and cycloheximide in U937 cells: pharmacological potential of nanoparticles. L. Mol. Cell. Biochem..

[69] Roskams T (2003). Oxidative stress and oval cell accumulation in mice and humans with alcoholic and nonalcoholic fatty liver disease. Am. J. Pathol..

[70] Nobili V (2013). Docosahexaenoic acid for the treatment of fatty liver: randomised controlled trial in children. Nutr. Metab. Cardiovasc. Dis..

[71] Jin DY, Chae HZ, Rhee SG, Jeang KT (1997). Regulatory role for a novel human thioredoxin peroxidase in NF-kappa-B activation. J. Biol. Chem..

[72] Lee K, Kerner J, Hoppel CL (2011). Mitochondrial Carnitine Palmitoyltransferase 1a (CPT1a) Is Part of an Outer Membrane Fatty Acid Transfer Complex. J. Biol. Chem..

[73] Sakamaki K, Tomonaga M, Tsukui K, Nagata S (1989). Molecular cloning and characterization of a chromosomal gene for human eosinophil peroxidase. J. Biol. Chem..

[74] Serviddio G (2011). Oxidation of hepatic carnitine palmitoyl transferase-I (CPT-1) impairs fatty acid beta-oxidation in rats fed a methionine-choline deficient diet. PLoS One..

[75] Glatz, J. F. C. & Luiken, J. J. F. P. Dynamic role of the transmembrane glycoprotein CD36 (SR-B2) in cellular fatty acid uptake and utilization. *J*. *Lipid*. *Res*. jlr.R082933 (2018). 10.1194/jlr.R082933 PMC6027920 29627764

[76] Wilson CG (2016). Hepatocyte-specific disruption of CD36 attenuates fatty liver and improves insulin sensitivity in HFD-fed mice. Endocrinology..

[77] Li BH, He FP, Yang X, Chen YW, Fan JG (2017). Steatosis induced CCL5 contributes to early-stage liver fibrosis in nonalcoholic fatty liver disease progress. Transl. Res..

[78] Zhang X (2014). CXCL10 plays a key role as an inflammatory mediator and a non-invasive biomarker of non-alcoholic steatohepatitis. J. Hepatol..

[79] Larter CZ (2007). Not all models of fatty liver are created equal: understanding mechanisms of steatosis development is important. J. Gastroenterol. Hepatol..

[80] Marchesini G (2016). EASL-EASD-EASO Clinical Practice Guidelines for the management of non-alcoholic fatty liver disease. J Hepatol..

[81] Italian Association for the Study of the Liver (AISF). AISF position paper on nonalcoholic fatty liver disease (NAFLD): Updates and future directions. *Dig*. *Liver*. *Dis*. **49**, 471–483 (2017). 10.1016/j.dld.2017.01.147 28215516

[82] Kirsch R (2003). Rodent nutritional model of non-alcoholic steatohepatitis: species, strain and sex difference studies. J. Gastroenterol. Hepatol..

[83] Park K (2018). Toxicity and tissue distribution of cerium oxide nanoparticles in rats by two different routes: single intravenous injection and single oral administration. Arch. Pharm. Res..

[84] Kumari M, Kumari SI, Grover P (2014). Genotoxicity analysis of cerium oxide micro and nanoparticles in Wistar rats after 28 days of repeated oral administration. Mutagenesis..

[85] Reichenbach V (2012). Prevention of Fibrosis Progression in CCl 4 -Treated Rats: Role of the Hepatic Endocannabinoid and Apelin Systems. J. Pharmacol. Exp. Ther..

[86] Burdge GC, Wright P, Jones AE, Wootton SA (2000). A method for separation of phosphatidylcholine, triacylglycerol, non-esterified fatty acids and cholesterol esters from plasma by solid phase extraction. Br. J. Nutr..

[87] Fisk HL, Wet AL, Childs CE, Burdge GC, Calder PC (2014). The use of gas chromatography to analyze compositional changes of fatty acid in rat liver tissue during pregnancy. J. Vis. Exp..

[88] Fernández-Galán E (2018). Validation of a routine gas chromatography mass spectrometry method for 2-hydroxyglutarate quantification in human serum as a screening tool for detection of idh mutations. J. Chromatogr. B. Analyt. Technol. Biomed. Life. Sci..

[89] Melgar-Lesmes P (2011). Hypoxia and proinflammatory factors upregulate apelin receptor expression in human stellate cells and hepatocytes. Gut.

